# The banana fruit Dof transcription factor MaDof23 acts as a repressor and interacts with MaERF9 in regulating ripening-related genes

**DOI:** 10.1093/jxb/erw032

**Published:** 2016-02-17

**Authors:** Bi-hong Feng, Yan-chao Han, Yun-yi Xiao, Jian-fei Kuang, Zhong-qi Fan, Jian-ye Chen, Wang-jin Lu

**Affiliations:** ^1^State Key Laboratory for Conservation and Utilization of Subtropical Agro-bioresources/Guangdong Key Laboratory for Postharvest Science, College of Horticultural Science, South China Agricultural University, Guangzhou 510642, PR China; ^2^College of Agriculture, GuangXi University, Nanning 530004, PR China

**Keywords:** Banana, Dof, ERF, fruit ripening, *Musa acuminate*, protein interaction, transcriptional regulation.

## Abstract

The DNA binding with one finger (Dof) proteins, a family of plant-specific transcription factors, are involved in a variety of plant biological processes. However, little information is available on their involvement in fruit ripening. We have characterized 25 *MaDof* genes from banana fruit (*Musa acuminata*), designated as *MaDof1*–*MaDof25*. Gene expression analysis in fruit subjected to different ripening conditions revealed that *MaDof*s were differentially expressed during different stages of ripening. *MaDof10*, *23*, *24*, and *25* were ethylene-inducible and nuclear-localized, and their transcript levels increased during fruit ripening. Moreover, yeast two-hybrid and bimolecular fluorescence complementation analyses demonstrated a physical interaction between MaDof23 and MaERF9, a potential regulator of fruit ripening reported in a previous study. We determined that MaDof23 is a transcriptional repressor, whereas MaERF9 is a transcriptional activator. We suggest that they might act antagonistically in regulating 10 ripening-related genes, including *MaEXP1*/*2*/*3*/*5*, *MaXET7*, *MaPG1*, *MaPME3*, *MaPL2*, *MaCAT*, and *MaPDC*, which are associated with cell wall degradation and aroma formation. Taken together, our findings provide new insight into the transcriptional regulation network controlling banana fruit ripening.

## Highlight

The banana transcriptional repressor MaDof23 and transcriptional activator MaERF9 may act antagonistically in regulating 10 ripening-related genes associated with cell wall degradation and aroma formation.

## Introduction

Transcriptional regulation of gene expression in higher plants plays a pivotal role in influencing or controlling many important biological processes, such as growth and development, signal transduction, and environmental stress responses (Riechmann and Ratcliffe, 2000). Transcription factors (TFs) are important regulators of gene expression that are able to bind to specific *cis*-regulatory elements in the promoter region, and activate or repress the transcription of their target genes (Wray *et al.*, 2003). In plants, about 7% of all genes encode putative TFs (Udvardi *et al.*, 2007). *Arabidopsis* genomes include at least 1533 TFs, and tomato (*Solanum lycopersicum*) at least 998 (Riechmann and Ratcliffe, 2000; Zhang *et al.*, 2010; Sato *et al.*, 2012). In banana (*Musa acuminata*), 3155 putative TFs have been identified among the 36 542 predicted protein-coding genes (D’Hont *et al.*, 2012). TFs are grouped into different families based on conserved structural domains. A wide range of TF families have been identified in plants, including WRKY, NAM/ATAF1/CUC2 (NAC), basic leucine zipper (bZIP), APETALA 2/ethylene-responsive element binding factor (AP2/ERF), basic helix-loop-helix, and Cys2(C2)His2-type zinc fingers (ZFs) (Lindemose *et al.*, 2013; Yamasaki *et al.*, 2013).

The DNA binding with one finger (Dof) proteins, which were first found in maize (*Zea mays*), are a family of plant-specific TFs of the ZF super-family, containing a highly conserved DNA binding Dof domain in the N-terminal region (Yanagisawa and Izui, 1993; Gupta *et al.*, 2015). The Dof domain is composed of 50–56 amino acid residues, structured as a C2/C2 ZF, mediating both DNA–protein and protein–protein interactions (Yanagisawa, 2002). The DNA binding sequence of all Dof proteins, except a pumpkin Dof protein (AOBP) that recognizes an AGTA DNA motif, includes the AAAG motif or its reverse-oriented sequence CTTT in the plant gene promoters (Diaz *et al.*, 2002; Yanagisawa, 2002; Gupta *et al.*, 2015). Since the first Dof TF was isolated from maize, Dof TFs have been identified in other plants, including *Arabidopsis*, rice (*Oryza sativa*), poplar (*Populus trichocarpa*), barley (*Hordeum vulgare*), bread wheat (*Triticum aestivum*), maize, sorghum (*Sorghum bicolor*), tomato, soybean (*Glycine max*), Chinese cabbage (*Brassica rapa* L. ssp. *pekinensis*), and potato (*Solanum tuberosum*) (Yanagisawa, 2002, 2004; Cai *et al.*, 2013; Guo and Qiu, 2013; Noguero *et al.*, 2013; Gupta *et al.*, 2015; Ma *et al.*, 2015; Venkatesh and Park, 2015). Moreover, genetic and molecular studies have suggested that Dof TFs are involved in many plant-specific physiological processes, such as light-responsiveness, tissue differentiation, seed development or germination, metabolic regulation, and phytochrome and phytohormone signalling (Noguero *et al.*, 2013; Gupta *et al.*, 2015). Recently, Dof TFs were reported to be involved in biotic and abiotic stress responses, possibly via the induction of various stress-responsive genes, such as *COR15*, *RD29A*, and *RD10* (Corrales *et al.*, 2014; Gupta *et al.*, 2015; Sasaki *et al.*, 2015). Given the diverse function of Dof TFs in plant biological processes, it is important to gain insights into their involvement in fruit ripening.

Banana is one of the most important fruit crops in the world, serving as part of a well-balanced human diet and a staple food for more than 400 million people in many tropical and subtropical countries (D’Hont *et al.*, 2012; Hölscher *et al.*, 2014). Unfortunately, banana is a typical climacteric fruit; once ripening is initiated, the shelf-life is limited owing to its rapid softening and susceptibility to diseases. This restricts the handling and transportation of the fruit, which can cause significant losses to both farmers and consumers (Bapat *et al.*, 2010; Shan *et al.*, 2012; Xiao *et al.*, 2013). Therefore, understanding the ripening mechanism of banana fruit is critical for improving its postharvest life. Several ripening-related TFs, including MADS-box (Elitzur *et al.*, 2010; Choudhury *et al.*, 2012; Liu *et al.*, 2013), EIN3/EIL (Mbéguié-A-Mbéguié *et al.*, 2008), NAC (Shan *et al.*, 2012), ERF (Xiao *et al.*, 2013), and LBD (Ba *et al.*, 2014a), as well as their involvement in the transcriptional control of ripening, have been reported. Fruit ripening is a complex and highly coordinated developmental process that involves dramatic changes in colour, flavour, aroma, texture, and nutritional content of the flesh, and is controlled by transcriptional regulatory networks involving several TFs (Giovannoni, 2004; Klee and Giovannoni, 2011; Martel *et al.*, 2011; Gapper *et al.*, 2013; Seymour *et al.*, 2013a, b; Cherian *et al.*, 2014). Whether extensive cooperation exists between the different types of fruit ripening-related TFs remains obscure.

There are 74 *Dof* genes in the banana genome (Plant Transcription Factor Database, http://planttfdb.cbi.pku.edu.cn), and 25 of them are differentially expressed during the ripening stages as revealed by an RNA sequencing analysis (D’Hont *et al.*, 2012). However, the regulatory mechanism of Dofs involved in banana fruit ripening is largely unknown. We have therefore isolated and characterized these 25 *Dof* genes, designated *MaDof1*–*MaDof25*, from banana fruit and analysed their expression patterns under three different ripening conditions. Moreover, we investigated the interactions of MaDofs with a previously identified ripening-related AP2/ERF TF, MaERF9, as well as their involvement in the transcriptional regulation of 11 ripening-related genes. These included genes responsible for softening, such as expansins (*MaEXP1*/*2*/*3*/*5*), xyloglucan endotransglycosylases (*MaXET7*), polygalacturonase (*MaPG1*), pectin methylesterase (*MaPME3*), pectate lyase (*MaPL2*), and galactosidase (*MaGAL*), and genes related to aroma formation, such as branched-chain amino acid transaminase (*MaCAT*) and pyruvate decarboxylase (*MaPDC*). Our findings expand our understanding of the transcriptional regulatory network of banana fruit ripening.

## Materials and methods

### Plant materials and treatments

Banana (*Musa acuminata*, AAA group, cv. Cavendish) fruit at the 70–80% plump stage were harvested from a commercial plantation near Guangzhou, south-eastern China. Three conditions, comprising natural ripening, ethylene-induced ripening (100 µL L^–1^ ethylene, 18h), and 1-methylcyclopropene (1-MCP)-delayed ripening (0.5 µl L^–1^ 1-MCP, 18h), were considered as described previously (Shan *et al.*, 2012). After each treatment, fruit were held at 22°C and 90% relative humidity until the production of climacteric ethylene and complete ripening. Sample taking, as well as the recording of ethylene production and fruit firmness at each sampling time, were described previously in Shan *et al.* (2012). All of the samples were frozen in liquid nitrogen and stored at −80°C for further use.

### *In silico* analysis

The whole genome sequence of *M. acuminata* was used to identify Dof TFs (http://banana-genome.cirad.fr/) (D’Hont *et al.*, 2012). On the basis of gene annotation and bioinformatics and RNA sequencing analyses, 25 *Dof* genes named *MaDof1* to *MaDof25* were identified. Total RNA was extracted using the method of Wan and Wilkins (1994) and the cDNA was obtained using PrimeScript^®^ RT Reagent Kit with gDNA Eraser (TaKaRa). The sequences of *MaDof1* to *MaDof25* were further verified by recloning and resequencing. Gene sequences were subjected to a homology search in the National Center for Biotechnology Information database. Multiple alignments were analysed by CLUSTALW (version 1.83) and GeneDoc software, and a phylogenetic tree of Dof proteins was constructed using the UPGMA method in the MEGA5.

### Gene expression analysis

Quantitative real-time PCR (qRT-PCR) were used to analyse gene expression. The sequences of all primers used for qRT-PCR are listed in Supplementary Table S1. All qRT-PCR analyses were normalized using the cycle threshold value of *MaRPS2* (ribosomal protein 2) as the reference gene (Chen *et al.*, 2011). qRT-PCR was carried out in a Bio-Rad CFX96 Real-Time PCR System using the SYBR^®^Green PCR Supermix Kit (Bio-Rad Laboratories) following the manufacturer’s instructions. The relative expression levels of target genes were calculated with the formula 2^-ΔΔCT^. Three independent biological replicates were used.

### Subcellular localization assay

The coding regions of *MaDof*s without the stop codon were amplified (primers are listed in Supplementary Table S1), and subcloned into the pEAQ vector containing the gene for green fluorescent protein (*GFP*; kindly supplied by Dr George P. Lomonossoff). The fusion constructs and the control GFP vector were electroporated into *Agrobacterium tumefaciens* strain GV3101 using the Gene Pulser Xcell^TM^ Electroporation System (Bio-Rad, CA). A tobacco (*Nicotiana benthamiana*) leaf infiltration assay for subcellular localization was performed (Sainsbury *et al.*, 2009). After infiltration, plants were incubated at 22°C with a 16h photoperiod for at least 48h before analysis. GFP fluorescence signals were observed with a fluorescence microscope (Zeiss Axioskop 2 Plus). All transient expression assays were repeated at least three times.

### Transcriptional activation analysis in yeast cells

The coding regions of *MaDof*s were cloned into the pGBKT7 [GAL4 DNA-binding domain (GAL4BD)] vector (Clontech, USA) to create the pGBKT7-MaDof constructs (primers are listed in Supplementary Table S1). pGBKT7-MaDofs, the positive control pGBKT7-53 + pGADT7-T, and the negative control pGBKT7 plasmids were transformed into the AH109 yeast strain using the lithium acetate method. The transformed strains were streaked onto plates with minimal medium without tryptophan (SD/−Trp) or without tryptophan, histidine, and adenine (SD/−Trp−His−Ade) plates, and the transactivation activity of each protein was evaluated according to their growth status and the activity of α-galactosidase (α-Gal).

### Yeast two-hybrid assay

A yeast two-hybrid (Y2H) assay was performed using the Matchmaker™ GoldYeast Two-Hybrid System (Clontech). The coding sequences of *MaDof23* and *MaERF9* were subcloned into the pGBKT7 or pGADT7 vector to fuse with the BD and activation domain (AD), respectively, to create the bait and prey (primers are listed in Supplementary Table S1). The bait and prey constructs were then co-transformed into yeast strain Gold Y2H using the lithium acetate method, and yeast cells were grown on DDO medium [minimal media double dropouts (SD/−Leu−Trp)] for 3 d. Transformed colonies were plated onto QDO medium [minimal media quadruple dropouts (SD/−Leu−Trp−Ade−His but containing 125 μm aureobasidin A)] containing 4mg mL^−1^ X-α-Gal to test the possible interaction between MaDof23 and MaERF9 according to their growth status and blue colour development.

### Bimolecular fluorescence complementation assay

To create constructs for a bimolecular fluorescence complementation (BiFC) assay, the full-length coding sequences, without their stop codons, of *MaDof23* in fusion with YNE and *MaERF9* in fusion with YCE were cloned into the pEAQ-HT vector (Sainsbury *et al.*, 2009). The resulting constructs were then electroporated into *A. tumefaciens* strain GV3101 and co-infiltrated into tobacco leaves. Infected tissues were analysed at 48h after infiltration. YFP fluorescence was captured using the Confocal Spectral Microscope Imaging System (Leica TCS SP5), with an argon blue laser at 488nm, a beam splitter for excitation at 500nm, and a spectral detector set between 515nm and 540nm. Primers used for generating the constructs are listed in Supplementary Table S1.

### Promoter isolation and analysis

Genomic DNA was extracted from banana leaves using the DNeasy Plant Mini Kit (Qiagen). The promoters of the 11 ripening-related genes, including *MaEXP1*/*2*/*3*/*5*, *MaXET7*, *MaPG1*, *MaPME3*, *MaPL2*, and *MaGAL* associated with cell wall degradation (Trivedi and Nath 2004; Sane *et al.*, 2007; Mbéguié-A-Mbéguié *et al.*, 2009; Asif *et al.*, 2014), and *MaCAT* and *MaPDC* associated with aroma formation (Yang *et al.*, 2011), were isolated using a Genome Walker Kit (Clontech) with nest PCR according to the manufacturer’s instructions (see specific primers in Supplementary Table S1). Conserved *cis*-element motifs of promoter were predicted using PLACE (http://www.dna.affrc.go.jp/PLACE/signalscan.html) and Plant-CARE (http://bioinformatics.psb.ugent.be/webtools/plantcare/html/) databases.

### Dual-luciferase reporter assay

To analyse the *in vivo* transcriptional activities of MaDof23 and MaERF9, the coding sequence of *MaDof23* or *MaERF9* was inserted into the constructed pBD vector driven by the 35S promoter with the translation enhancer Ω sequence as the effector. The double reporter vector included a native GAL4-LUC (Firefly luciferase), and an internal control REN (*Renilla* luciferase) driven by a 35S promoter, which was modified based on the pGreenII 0800-LUC reporter vector (Hellens *et al.*, 2005). GAL4-LUC contains five copies of the GAL4 binding element and 35S promoter or a minimal TATA region of the 35S promoter of cauliflower mosaic virus (CaMV), and these sequences are located upstream of the LUC.

To assay the binding activity of MaDof23 or MaERF9 to the promoters of the 11 ripening-related genes mentioned above, the promoters were cloned into a pGreenII 0800-LUC double-reporter vector, while *MaDof23* or *MaERF9* was cloned into the pEAQ vector as the effectors. All primers used for generating constructs for the transient expression assay are listed in Supplementary Table S1.

The constructed effector and reporter plasmids were co-transformed into tobacco leaves by *A. tumefaciens* strain GV3101. After 2 d, LUC and REN luciferase activities were measured using a Dual-Luciferase Assay kit (Promega) on the Luminoskan Ascent Microplate Luminometer (Thermo). The results were calculated using the ratio of LUC to REN. At least six biological repeats were assayed for each combination.

### Statistical analysis

The study was carried out using a completely randomized design. In figures, data have been plotted as means ± standard errors (SE). Statistical comparisons of the mean values was performed using a one-way ANOVA, followed by Duncan’s multiple range test at the 0.05 or 0.01 confidence levels using the DPS7.05 software (Zhejiang University, Hangzhou, China). A complete linkage hierarchical clustering of the 25 *MaDof* genes in banana fruit pulp during ethylene-induced ripening was generated using the MeV 4.9 clustering algorithm according to their gene expression levels.

## Results

### Sequence analysis of 25 *Dof* genes and their deduced protein products in banana fruit

**Fig. 1.**
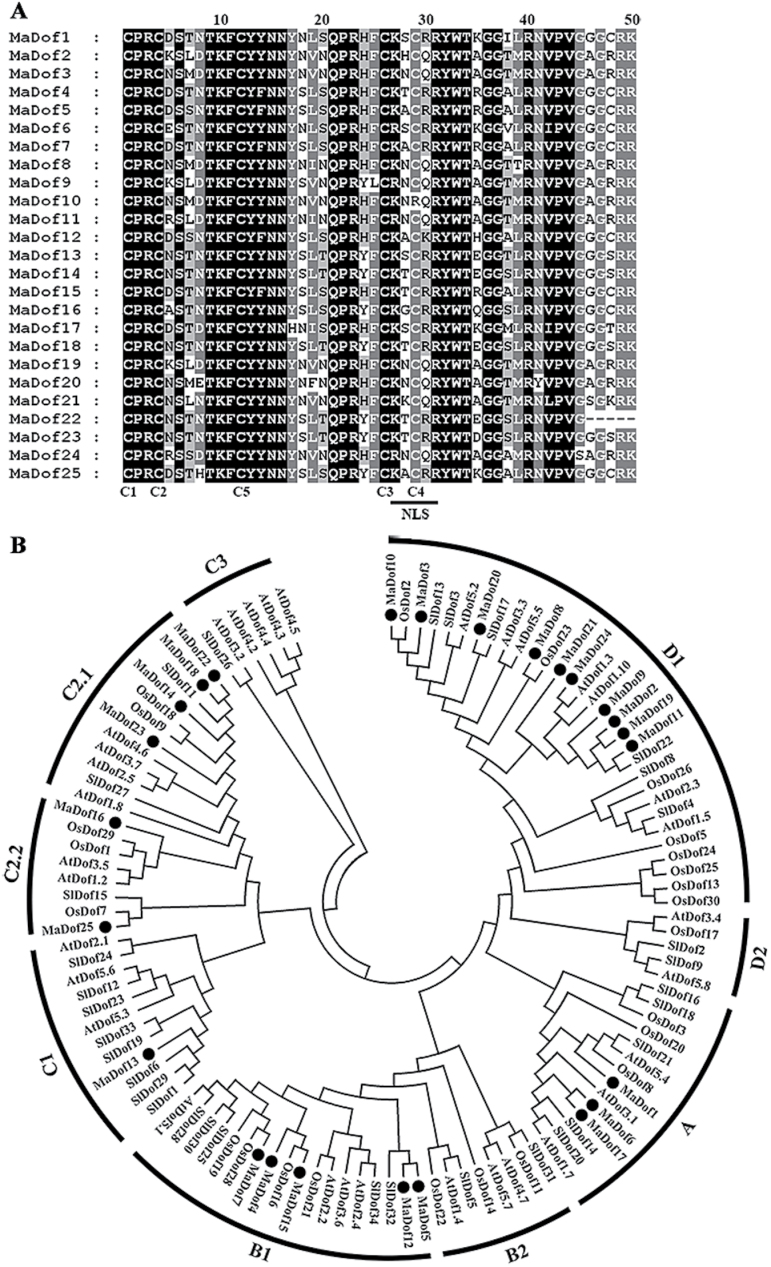
(**A**) Multiple sequence alignment of the conserved Dof domain of 25 MaDof proteins. Identical and similar amino acids are indicated by black and grey shading, respectively. Gaps were introduced to optimize alignment. The NLS motif, and conserved cysteine residues (C1–C5) within the Dof domain are indicated in the alignment. (**B**) Phylogenetic tree of Dofs. Banana MaDofs were aligned with the Arabidopsis, rice, and tomato Dof family. The multiple alignment was made using ClustalW, and the phylogenetic tree was constructed with MEGA 5.0 using a bootstrap test of phylogeny with minimum evolution test and default parameters.

Twenty-five *Dof* genes named *MaDof1*–*MaDof25* were isolated from banana fruit. The deduced amino acid residues of *MaDof1*–*M*aDof25 varied from 182 to 511 amino acids with the isoelectric point (*p*I) from 5.29 to 9.93, and the molecular mass from 19.43kDa to 54.68kDa (Supplementary Table S2). The sequence similarities of the MaDofs varied from 6.4% (MaDof4 and MaDof7) to 72.6% (MaDof1 and MaDof6) (Supplementary Table S3). Alignments of MaDofs showed a highly conserved DBD, designated the Dof domain and located in the N-terminal region, that consisted of 50–56 amino acid residues structured as a C2/C2 ZF (Fig. 1A), which is a defining character of the Dof TF family (Cai *et al.*, 2013). A nuclear localization signal (NLS), overlapping partly with the highly conserved Dof domain (Krebs *et al.*, 2010; Noguero *et al.*, 2013), has also been identified in all MaDof proteins (Fig. 1A), suggesting the nuclear localization of MaDof proteins.

Phylogenetic analysis of MaDofs and Dofs from other plants, including Arabidopsis, rice, and tomato, revealed that MaDof sequences were clustered into six of the nine subfamilies of Dof proteins (Cai *et al.*, 2013) (Fig. 1B). These six subfamilies, including A, B1, C1, C2.1, C2.2, and D1, contained 3, 5, 1, 4, 2, and 10 MaDofs, respectively (Fig. 1B).

To gain more insight into the diversity of the 25 MaDof proteins selected in this study, we analysed putative motifs using the program MEME. Fifteen distinct motifs were identified, and annotated by InterProScan (Supplementary Fig. S1). Motif 1, uniformly observed in all of the MaDof proteins, is the conserved Dof domain. As expected, most of the closely related members in the phylogenetic tree had common motif compositions, suggesting functional similarities among the Dof proteins within the same subfamily (Supplementary Fig. S2).

### Differential expression of *MaDof* genes in fruit pulp during ripening

**Fig. 2.**
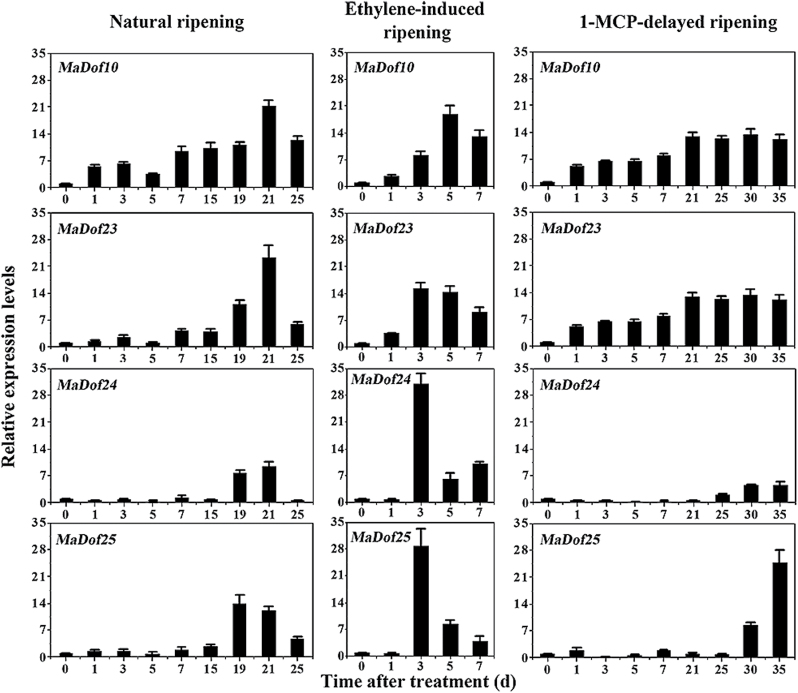
Expressions of *MaDof10*, *23*, *24*, and *25* in pulp during three ripening behaviours: natural (control), ethylene-induced, and 1-MCP-delayed ripening. The expression levels of each gene are represented as a ratio relative to the harvest time (0 d of control), which was set at 1. Each value represents the mean ± SE of three biological replicates. The physiological data related to fruit ripening and softening, including changes in fruit firmness and ethylene production in banana fruit subjected to three different ripening conditions, have been described in Shan *et al.* (2012).

To investigate the possible role of *MaDof* genes in relation to banana fruit ripening, we first detected their mRNA accumulations in fruit pulp during ethylene-induced ripening using qRT-PCR. We observed differential expression patterns of *MaDof*s during banana fruit ripening. Among the 25 *MaDof* genes, *MaDof2*, *3*, *10*, *20*, *23*, *24*, and *25* were obviously induced after ethylene treatment, reaching their maximum level at 3 or 5 d of storage, and then decreasing thereafter (Supplementary Fig. S3; Fig. 2). In contrast, transcripts of the other 18 *MaDof*s exhibited only slight changes (Supplementary Fig. S3). Based on the phylogenetic tree and expression profiles of *MaDof*s, we selected four *MaDof* genes from three different subfamilies, that is, *MaDof10* and *24* (subfamily D1), *MaDof23* (subfamily C2.1), and *MaDof25* (subfamily C2.2), and measured their expressions in fruit pulp under two different ripening conditions: natural and 1-MCP-delayed ripening. Their transcript levels in fruit pulp under natural or 1-MCP-delayed ripening also clearly increased following ripening (Fig. 2).

### Nuclear localization of MaDof10, 23, 24, and 25

**Fig. 3.**
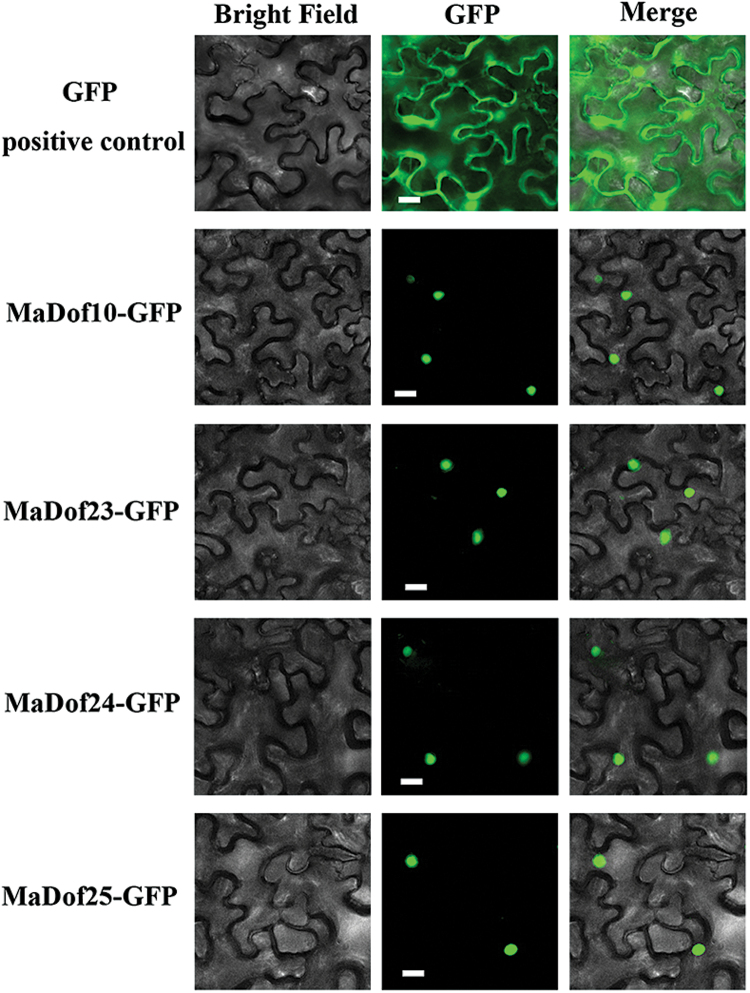
Subcellular localization of MaDof10, 23, 24, and 25 in tobacco leaves. MaDof10, 23, 24, and 25 fused with the GFP or GFP positive control were infiltrated into tobacco leaves via *A. tumefaciens* strain GV3101. After 48h of infiltration, GFP fluorescence signals were visualized using a fluorescence microscope. Merge indicates a digital merge of bright field and fluorescent images. Images were taken in a dark field for green fluorescence, while the outline of the cell and the Merged images were photographed in a bright field. Scale bars, 25 μm.

Potential NLS sequences were predicted for MaDof10, 23, 24, and 25 based on the sequence analysis (Fig. 1A). To validate the subcellular localizations of MaDof10, 23, 24 and 25, the full-length coding sequences of these proteins were fused in frame with GFP. The fluorescence from the transient expression of these constructs in tobacco leaf epidermal cells was localized exclusively in the nucleus (Fig. 3). By contrast, fluorescence from the GFP-only control was distributed throughout the entire cell. We also observed similar localization when GFP-MaDof10, 23, 24, or 25 were transiently expressed in tobacco BY2 protoplasts (Supplementary Fig. S4).

### Transcriptional activation ability of MaDof10, 23, 24, and 25 proteins in yeast

**Fig. 4.**
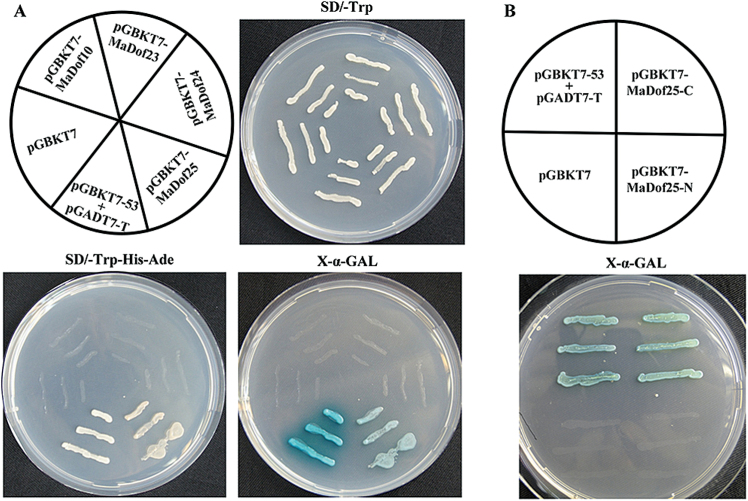
Transcriptional activation of MaDof10, 23, 24, and 25 in yeast. The coding regions of *MaDof10*, *23*, *24*, and *25*, as well as C- and N-terminal derivatives of *MaDof25*, were cloned into the pGBKT7 (GAL4BD) vector to create the pGBKT7-MaDof10, 23, 24, 25, 25-N, and 25-C constructs. All of the constructs mentioned above, together with the positive control (pGBKT7-53 + pGADT7-T) and negative control (pGBKT7) were transformed into yeast strain AH109. Yeast clones transformed with different constructs were grown on SD plates without tryptophan (SD/−Trp) or without tryptophan, histidine, and adenine but containing 125 μM aureobasidin A (SD/−Trp−His−Ade) for 3 d at 30°C. Transcription activation was monitored by the detection of yeast growth and an α-Gal assay.

To investigate the abilities of MaDof10, 23, 24, and 25 to activate transcription, we performed a transient expression assay using a GAL4-responsive reporter system in yeast cells. The full-length coding regions of *MaDof10*, *23*, *24*, and *25* were fused to the GAL4BD to generate pGBKT7-MaDof10, 23, 24, and 25 fusion plasmids (Fig. 4). The plasmids were then transformed into yeast strain AH109 and the transformants were assayed for their ability to activate transcription from the GAL4 upstream activation sequence and to promote yeast growth in medium lacking Trp, His, and Ade. The transformants containing pGBKT7-53 + pGADT7-T and pGBKT7 vectors (empty) were used as positive and negative controls, respectively. As shown in Fig. 4, the transformed yeast cells harbouring pGBKT7-MaDof25 and pGBKT7-53 + pGADT7-T (positive control) grew well in SD/−Trp−His−Ade and showed α-Gal activity, whereas cells containing pGBKT7-MaDof10, -MaDof23, -MaDof24, or pGBKT7 alone (negative control) showed no α-Gal activity. To further identify the transcriptional activation domain of MaDof25, the N- and C-terminal regions of *MaDof25* were fused to the GAL4BD. The yeast cells harbouring pGBKT7-MaDof25-C showed α-Gal activity (Fig. 4). These results indicate that the MaDof25 C-terminal region is able to activate transcription.

### Interaction between MaDof23 and MaERF9

**Fig. 5.**
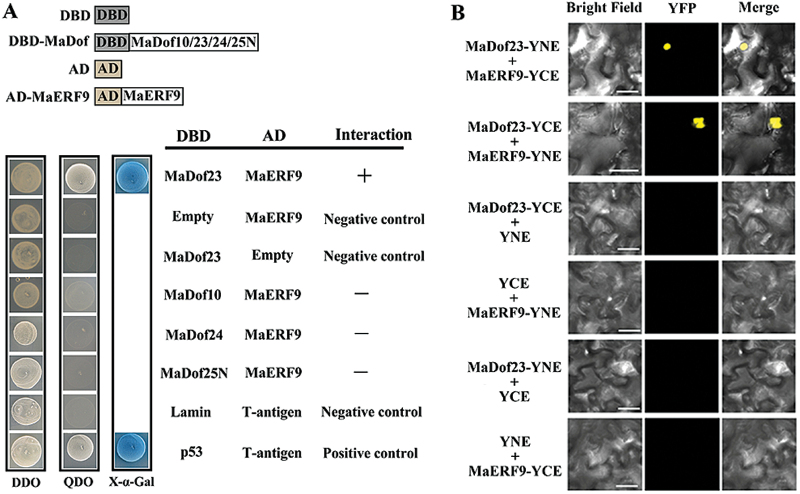
*In vitro* and in *vivo* interaction between MaDof23 and MaERF9. (**A**) A Y2H assay for the interaction between MaDof23 and MaERF9. The coding regions of MaDof10, 23, 24, and 25-N were fused with pGBKT7 (BD) and the coding region of MaERF9 with pGADT7 (AD) vectors as indicated, and co-transformed into the yeast strain Gold Y2H. The ability of yeast cells to grow on QDO medium (SD/−Leu−Trp−Ade−His but containing 125 μm aureobasidin A), and to turn blue in QDO medium containing 4mg mL^−1^ X-α-Gal, was scored as a positive interaction. (**B**) BiFC in tobacco leaf epidermal cells showing the interaction between MaDof23 and MaERF9 in living cells. MaDof23 and MaERF9 were fused with the N-terminus of YFP (YNE) or the C-terminus of YFP (YCE), as indicated, and co-transfected into *N. benthamiana* leaves by *A. tumefaciens* infiltration. Expressions of MaDof23 or MaERF9 alone were used as negative controls. YFP indicates fluorescence of YFP; Merge indicates a digital merge of bright field and fluorescent images. Scale bar, 30 μm.

In a previous study, we reported that MaERF9 might act as transcriptional activator in the regulation of fruit ripening by activating the expression of ethylene biosynthetic genes (Xiao *et al.*, 2013). Because the canonical recognition sequence for Dof TFs is very short, it is highly likely that they function in association with other TFs and in combination generate the necessary promoter specificity (Noguero *et al.*, 2013). Thus, we investigated whether MaDof10, 23, 24, and 25 can interact with MaERF9. First we used the GAL4 transcription activation-based Y2H system. Full-length sequences of *MaDof10*, *23*, and *24* and the N-terminus of *MaDof25* were fused with the GAL4BD (BD-MaDof10, 23, 24, and 25-N), and MaERF9 was ligated with the activation domain (AD-MaERF9), to create the bait and prey, respectively, given that MaERF9 has transcription-activating activity in yeast (data not shown). As shown in Fig. 5A, co-expression of BD-MaDof23 with AD-MaERF9 resulted in strong activation of α-Gal activity, indicating that only MaDof23 interacted with MaERF9 in yeast cells, whereas MaDof10, 24, and 25 did not.

To further substantiate that MaDof23 interacts with MaERF9 in plant cells, we examined their interaction by BiFC assay. Both MaDof23 and MaERF9 were fused to the N-terminal 174-amino acid portion of yellow fluorescent protein (YFP) as well as the C-terminal 66-amino acid portion of YFP, in the pEAQ vector. The corresponding constructs were co-transfected into *N. benthamiana* leaves by *A. tumefaciens* infiltration. As shown in Fig. 5B, co-expression of MaDof23-YNE and MaERF9-YCE, or MaDof23-YCE and MaERF9-YNE reconstituted a functional YFP in the nucleus, whereas co-expression with the negative control combinations failed to generate YFP fluorescence. These data together confirm that MaDof23 physically interacts with MaERF9.

### *In vivo* transcriptional activities of MaDof23 and MaERF9

**Fig. 6.**
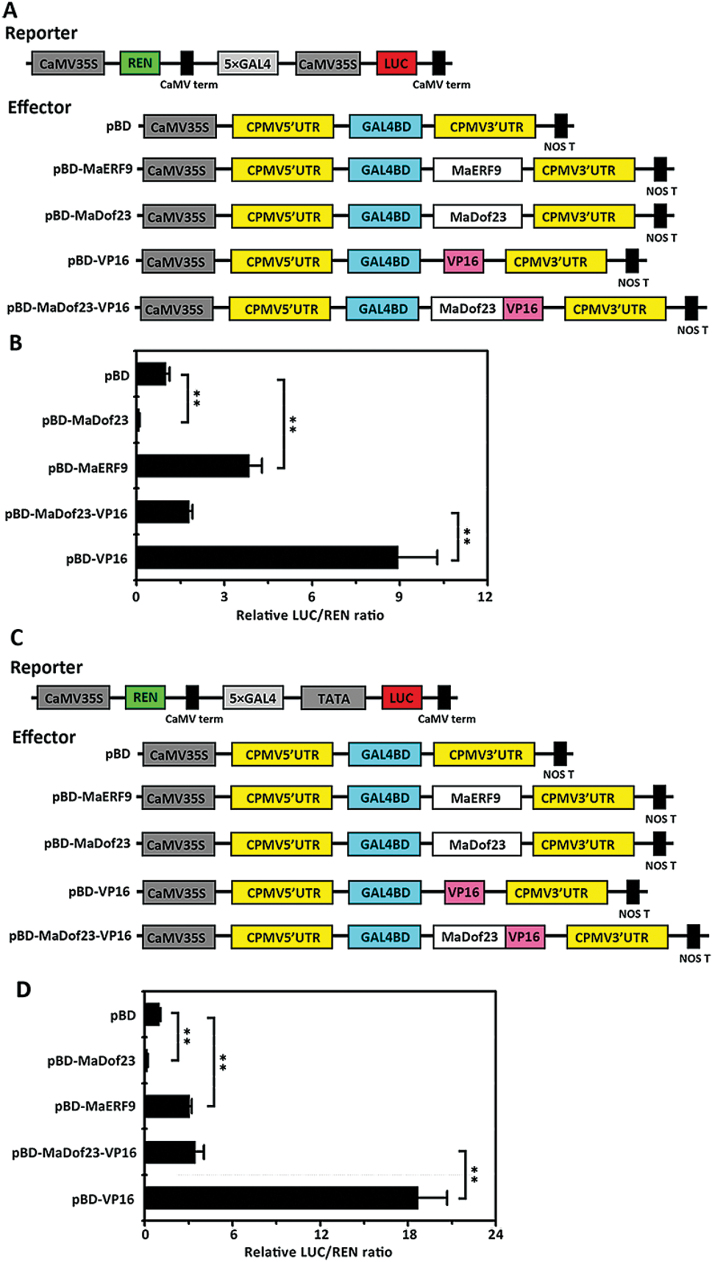
Transcriptional repression or activation ability of MaDof23 or MaERF9 in tobacco leaves. (**A** and **C**) Diagram of the various constructs used in this assay. The double-reporter plasmids contained LUC luciferase fused with 5 × GAL4 and CaMV35S or 5 × GAL4 and the minimal TATA region of CaMV35S, and REN luciferase driven by CaMV35S. The effector plasmids contained the MaDof23 or MaERF9 genes fused to GAL4BD driven by the CaMV35S. pBD was used as a negative control, while the GAL4BD fused with VP16 activation domain was used as positive control. (**B** and **D**) The dual REN/LUC reporter and effectors were co-transformed into tobacco leaves using *A. tumefaciens* strain GV3101. After 48h of infiltration, LUC and REN luciferase activities were assayed, and the transcription repression or activation ability of MaDof23 or MaERF9 is indicated by the ratio of LUC to REN. The ratio of LUC to REN of the pBD vector was used as a calibrator (set as 1). Each value represents the means of six biological replicates, and vertical bars represent the SE. Asterisks indicate a statistically significant difference compared with pBD by one-way ANOVA; *P < 0.05, **P < 0.01. This figure is available in colour at *JXB* online.

To investigate the transcriptional activities of MaDof23 and MaERF9 *in vivo*, we performed a dual-luciferase assay. The double-reporter vector contained 5 × GAL4 and CaMV35S fused to LUC and REN driven by CaMV35S, and the effector plasmids contained the *MaDof23* or *MaERF9* gene fused to the GAL4BD driven by the CaMV35S (Fig. 6A). These constructs were co-expressed in tobacco leaves by *Agrobacterium* transfection. As shown in Fig. 6B, compared with the GAL4BD (empty, pBD) control, pBD-MaDof23 significantly repressed the expression of the LUC reporter, and the LUC to REN ratio of MaDof23 was 11.9% that of the pBD control. pBD-MaERF9, together with the transcriptional activator control pBD-VP16, strongly activated the LUC reporter gene, and the LUC to REN ratio of MaERF9 was 1.37-fold higher than that of the pBD control (Fig. 6B). However, the activation of pBD-VP16 was significantly inhibited by MaDof23 (pBD-MaDof23-VP16) (Fig. 6B), in a manner similar to that of the repression domain of ERF3 (Ohta *et al.*, 2001). Similarly, when the LUC reporter gene was fused with 5 × GAL4 and the minimal TATA region, we also observed that pBD-MaDof23 remarkably repressed, and pBD-MaERF9 activated, the expression of the LUC reporter compared with pBD alone (Fig. 6C, D). The activation of pBD-VP16 was also drastically impaired by MaDof23 (Fig. 6D). These *in vivo* data demonstrate that MaDof23 may act as a transcriptional repressor, and MaERF9 as a transcriptional activator.

### Antagonistic action of MaDof23 and MaERF9 in transcriptionally regulating 10 ripening-related genes

**Fig. 7.**
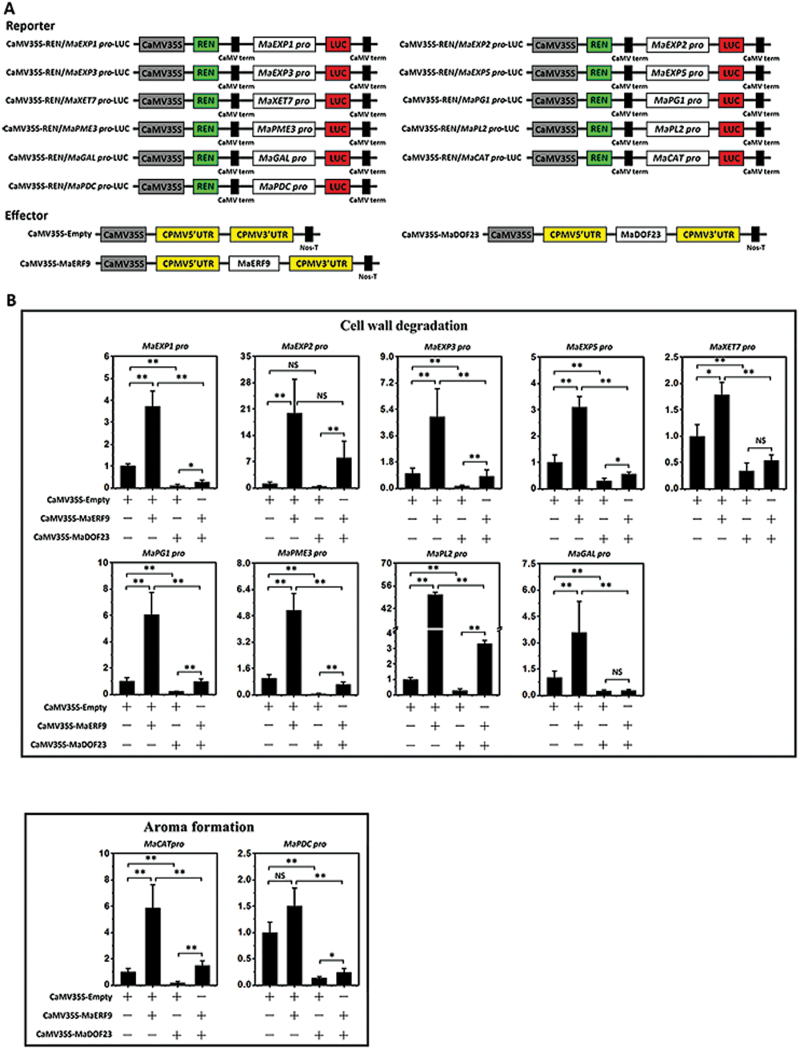
Antagonistic action of MaDof23 and MaERF9 in transcriptional regulation of 10 ripening-related genes including *MaEXP1*/*2*/*3*/*5*, *MaXET7*, *MaPG1*, *MaPME3*, and *MaPL2* associated with cell wall degradation; and *MaCAT* and *MaPDC* associated with aroma formation. (**A**) Diagram of the various constructs used in this assay. The double-reporter plasmids contained the promoter of the 11 ripening-related genes fused to LUC luciferase and REN luciferase driven by CaMV35S. The effector plasmids contained MaDof23 or MaERF9 driven by CaMV35S. (**B**) The reporter and effector vectors were co-introduced into tobacco leaves using *A. tumefaciens* strain GV3101. After 48h of infiltration, LUC and REN luciferase activities were assayed, and the repression or activation of MaDof23 or MaERF9 to the promoter was showed by the ratio of LUC to REN. The ratio of LUC to REN of the empty vector plus promoter vector was used as a calibrator (set as 1). Each value represents the mean ± SE of six biological replicates. Asterisks indicate a statistically significant difference by one-way ANOVA; *P < 0.05, **P < 0.01. + or – indicates that the effector was present or absent in the combinations. This figure is available in colour at *JXB* online.

It is well documented that Dof TFs preferentially bind to the core sequence 5′-(T/A)AAAG-3′ (or its reversibly orientated sequence, CTTT) and the and ERF TFs to the GCC-box in their target promoters (Hao *et al.*, 1998; Diaz *et al.*, 2002; Yanagisawa 2002). Sequence analysis identified the (T/A)AAAG elements and GCC-box in the promoters of all 11 ripening-related genes, including *MaEXP1*/*2*/*3*/*5*, *MaXET7*, *MaPG1*, *MaPME3*, *MaPL2*, *MaGAL*, *MaCAT*, and *MaPDC* (Supplementary Fig. S5), suggesting that these 11 ripening-related genes might be direct targets of MaDof23 and MaERF9. Moreover, the physical interaction between MaDof23 and MaERF9 led us to investigate whether they might mutually interfere in regulating target genes. To this end, we performed additional transient expression assays using the dual-luciferase reporter system. In this experiment, a dual-luciferase reporter plasmid harbouring the promoters of 11 ripening-related genes fused to LUC, together with an effector plasmid expressing MaDof23 or MaERF9 under the control of the CaMV35S promoter (Fig. 7A), were transiently co-expressed in the leaves of *N. benthamiana*. Compared with the control, co-expression of MaDof23 with each of the 11 promoters significantly repressed the LUC to REN ratio. In contrast, co-expression of MaERF9 enhanced the LUC to REN ratio (Fig. 7B), suggesting that MaDof23 trans-represses and MaERF9 trans-activates these ripening-related genes. Moreover, except for *MaGAL*, the trans-activation of MaERF9 or the trans-repression of MaDof23 of the other 10 gene promoters was impaired when MaDof23 and MaERF9 were co-expressed (Fig. 7B). Collectively, these data reveal that MaDof23 and MaERF9 might act antagonistically in the transcriptional regulation of ripening-related genes.

## Discussion

Dofs constitute a large family of TFs that are associated with various biological processes unique to plants. The identification of *Dof* genes and their evolution have been reported in *Arabidopsis* and several crop species, such as rice (Lijavetzky *et al.*, 2003), bread wheat (Shaw *et al.*, 2009), sorghum (Kushwaha *et al.*, 2011), tomato (Cai *et al.*, 2013), and Chinese cabbage (Ma *et al.*, 2015). However, little information is available on Dof TFs in fruits of substantial economic interest. We have identified 25 *MaDof* genes in banana based on the *Musa* genome (D’Hont *et al.*, 2012). Similar to the previous reports (Cai *et al.*, 2013; Ma *et al.*, 2015), alignment of the 25 MaDof proteins showed that they shared a highly conserved Dof domain in the N-terminus (Fig. 1A), although they were highly diverse in length, ranging from 182 to 511 amino acids. The lowest similarity observed was 6.4% while the highest similarity observed was 72.6% (Supplementary Table S2, S3). The 25 MaDofs fell into six out of the nine different subgroups of the previously characterized Dof proteins (Cai *et al.*, 2013; Ma *et al.*, 2015) (Fig. 1B). In addition, except for the Dof domain, another 14 distinct motifs were identified, and were differentially distributed in different subgroups (Supplementary Figs S1 and S2), suggesting that different subgroups of Dof proteins may exhibit diverse functions (Cai *et al.*, 2013; Ma *et al.*, 2015).

The involvement of Dof TFs in various biological processes has been reported, including seed storage protein accumulation, carbon and nitrogen metabolism, light responses, plant morphology, guard cell development, seed germination, and stress responses (Noguero *et al.*, 2013; Gupta *et al.*, 2015). In this study, *MaDof*s exhibited differential expression patterns during banana fruit ripening. Among the 25 *MaDof* genes, *MaDof10*, *23*, *24*, and *25* were induced during ripening. Further, their expression was also upregulated following ethylene treatment (Fig. 2; Supplementary Fig. S3), indicating that their products might be associated with banana fruit ripening. This is the first evidence of a possible role for Dof TFs in fruit ripening. Dof proteins are suggested to act as TFs based on their nuclear localization and DNA-binding activity (Noguero *et al.*, 2013; Gupta *et al.*, 2015). Consistent with their role as TFs, all the 25 deduced MaDofs have basic amino acid regions that potentially serve as NLSs to target proteins to the nucleus (Fig. 1A). Nuclear localization was effectively confirmed by transient expression of MaDof10, 23, 24, and 25 in tobacco BY-2 protoplasts (Fig. 3). Interestingly, MaDof25 possessed transcriptional activation properties in yeast, while MaDof23 exhibited transcriptional repression activity *in vivo* (Figs 4 and 6), revealing that MaDofs may act as transcriptional activators or repressors involved in fruit ripening.

Over the last few years, more and more ripening-related TFs, such as MADS-box/RIN, NAC/NOR, CNR, and ERF, have been reported in various fruits (Elitzur *et al.*, 2010; Yin *et al.*, 2010; Klee and Giovannoni 2011; Seymour *et al.*, 2011; Karlova *et al.*, 2014). Extensive efforts have made to identify their downstream targets, especially for tomato MADS-box/RIN, which controls the expression of a wide range of ripening-related genes, including those involved in ethylene biosynthesis and perception, cell wall metabolism, carotenoid formation, aroma formation, and ATP generation (Seymour *et al.*, 2013a, b). ERF TFs are also proposed to modulate fruit ripening by regulating cell wall-modifying genes (Yin *et al.*, 2010). Many genes expressed in relation to banana ripening, particularly with regard to ethylene biosynthesis, pulp softening, and aroma development, have been isolated and characterized, including the *MaACS* and *MaACO* gene family (Huang *et al.*, 2006; Inaba *et al.*, 2007), associated with ethylene biosynthesis; *MaEXP*s, *MaXET/XTH*s, *MaPME*, *MaPG*s, and *MaGAL*s (Trivedi and Nath 2004; Sane *et al.*, 2007; Mbéguié-A-Mbéguié *et al.*, 2009; Asif *et al.*, 2014), associated with cell wall degradation; and *MaCAT* and *MaPDC*, associated with aroma formation (Yang *et al.*, 2011). In our recent reports, we demonstrated that MaERFs, MaLBDs, and MaBSD1 TFs are involved in fruit ripening, at least in part via transcriptional regulation of *MaACS*, *MaACO*, *MaEXP1*, and *MaEXP2* (Xiao *et al.*, 2013; Ba *et al.*, 2014a, b). However, the transcriptional regulatory networks of these ripening-related genes are still largely unknown. In the present work, we isolated the promoters of 11 ripening-related genes, including *MaEXP1*/*2*/*3*/*5*, *MaXET7*, *MaPG1*, *MaPME3*, *MaPL2*, *MaGAL*, *MaCAT*, and *MaPDC* (Supplementary Fig. S5). We showed that MaERF9 is an activator and MaDof23 a repressor of these ripening-related genes (Figs 6 and 7), implying that MaERF9 and MaDof23 may be playing opposite roles or acting antagonistically in the regulation of banana fruit ripening. Whether MaERF9 and MaDof23 regulate these 11 ripening-related genes via direct binding to the (T/A)AAAG elements or GCC-box in their promoters needs to be further investigated.

Often, many different TFs regulate the expression of a particular gene. Therefore, fine-tuning of these different types of TFs is necessary. This is thought to occur through the formation of enhanceosome or repressosome complexes that affect protein–protein and protein–DNA interactions (Martinez and Rao, 2012; Xu *et al.*, 2013). Although multiple ripening-related TFs have been explored, only a few studies have shown them to cooperate in fruit ripening. Recently, RIN was found to interact with FUL1 and FUL2 (Shima *et al.*, 2013), and *in vitro* protein binding assays showed that FUL homologs, RIN, and tomato AGAMOUS-LIKE1 form DNA binding complexes (Fujisawa *et al.*, 2014). However, whether the tetramer complex of these MADS-box proteins increases or decreases the transcriptional activation of ripening-related genes is largely unknown. Previous studies suggested that Dof TFs form heterodimers with other TFs, such as bZIP, MYB, ZFP, and TCP, and are associated with hormone signalling, stress responses, endosperm development, floral organ abscission, and germination (Zhang *et al.*, 1995; Washio 2003; Diaz *et al.*, 2005; Wei *et al.*, 2010; Rueda-Romero *et al.*, 2012). In the present investigation, we demonstrated that the transcriptional repressor MaDof23 physically interacts with the transcriptional activator MaERF9 (Fig. 5). We found that the trans-activation of MaERF9 or the trans-repression of MaDof23 to its target gene promoters was impaired by their interaction (Fig. 7). Interestingly, ethylene induced *MaDof23*, but MaDof23 in turn repressed ripening-related genes (Figs 2 and 7). Similarly, SlZFP2 is induced by abscisic acid (ABA), but represses ABA biosynthesis during fruit development by directly suppressing the ABA biosynthesis genes, suggesting that S1ZEP2 acts as a repressor to fine-tune ABA biosynthesis during fruit development (Weng *et al.*, 2015). It could be speculated that MaDof23 plays a role in fine-tuning the regulation of banana fruit ripening by negatively regulating the expression of ripening-related genes, possibly balancing the inductive effects of transcriptional activators such as *MaERF9*. In addition, it is possible that MaDof23 and MaEFRF9 competitively bind to the promoters of ripening-related genes via an unknown *cis*-element, leading to the antagonistic action between MaDof23 and MaERF9; however, this speculation need further investigation. Nevertheless, the interaction between MaDof23 and MaERF9 raises an interesting question of why plants use such an antagonistic interaction in fruit ripening. Given that Dofs are one of the largest TF families in plants, efforts should be made to elucidate whether other banana fruit MaDof TFs interact with MaERF9 to form enhanceosomes that synergistically regulate ripening-related genes.

In summary, we identified 25 banana fruit *MaDof* genes and found they were differentially expressed in pulp tissues during fruit ripening. Transient expression assays indicated that MaDof23 is a transcriptional repressor and physically interacts with MaERF9, a regulator of fruit ripening in banana. These proteins may act antagonistically to regulate the 10 ripening-related genes associated with cell wall degradation and aroma formation. Thus, our results have revealed that MaDofs regulate fruit ripening at least in part by the transcriptional regulation of specific ripening-related genes. Our findings provide new insights into the transcriptional regulatory network of fruit ripening in banana.

## Supplementary data

Supplementary materials are available at *JXB* online.

Table S1. Summary of primers used in this study.

Table S2. Related information for each *MaDof* gene.

Table S3. Sequence similarities among the different *MaDof* genes.

Fig. S1. Multilevel consensus sequence identified by MEME among banana fruit MaDof proteins.

Fig. S2. Schematic distribution of conserved motifs in banana fruit MaDofs identified by MEME.

Fig. S3. Hierarchical clustering analysis of 25 *MaDof* expression profiles in ethylene-treated banana fruit pulp.

Fig. S4. Subcellular localization of MaDof10, 23, 24, and 25 in tobacco BY-2 protoplasts.

Fig. S5. Nucleotide sequences of the promoters of 11 ripening-related genes.

Supplementary Data
