# Supplementary material for: The banana fruit Dof transcription factor MaDof23 acts as a repressor and interacts with MaERF9 in regulating ripening-related genes
Source: J Exp Bot. 2016 Feb 17;67(8):2263–75. doi: 10.1093/jxb/erw032 (PMC4809287; doi:10.1093/jxb/erw032)
Supplement: Supplementary Data [file supp_67_8_2263__index.html]

The banana fruit Dof transcription factor MaDof23 acts as a repressor and interacts with MaERF9 in regulating ripening-related genes — The banana fruit Dof transcription factor MaDof23 acts as a repressor and interacts with MaERF9 in regulating ripening-related genes — Supplementary Data 

# The banana fruit Dof transcription factor MaDof23 acts as a repressor and interacts with MaERF9 in regulating ripening-related genes

## Supplementary Data

Data files

- supplementary\_figures\_S1\_S4\_tables\_S1\_S2.pdf - Supplementary Data
- supplementary\_table\_S3.xlsx - Supplementary Data
