# Supplementary material for: The banana fruit Dof transcription factor MaDof23 acts as a repressor and interacts with MaERF9 in regulating ripening-related genes
Source: J Exp Bot. 2016 Feb 17;67(8):2263–75. doi: 10.1093/jxb/erw032 (PMC4809287; doi:10.1093/jxb/erw032)
Supplement: Supplementary Data [file supp_erw032_supplementary_figures_S1_S4_tables_S1_S2.pdf]

Supplementary data

Table S1. Summary of primers used in this study.

Assay	Gene	Forward(5'-3')	Reverse(5'-3')
RT-qPCR	*MaDof1*	ATCTCCATCTCCAATCCCAACCC	GCGGACCGTCTGATCTTCGA
	MaDof2	CTCCAGACTCTCCATCACCCTA	CATTCTTCACCATGTGTCCTTCC
	MaDof3	GGAAATGGCACCGTCCTTAGAT	AGAGGGAAAGGGAGTGTTGCTG
	MaDof4	CAGCACTATGAACCGAGTCCTT	AGAAATCTGTCGTCCAACCTCC
	MaDof5	GAACTTGAGGCTTCATCAGCACAT	CACTACCTTCTCCAATAAACGACG
	MaDof6	TCCATCTCCAACCCCAACCCT	GTCCGCCCAATGGCTCTGAT
	MaDof7	AACTACTCCTTGTCGCAGCCC	AAGCATCAGTGCCGCCAAAGAG
	MaDof8	CGTAAAAGTAAGCACTCTGCATCAC	TCCCTGTCTTCTCCTCACCCT
	MaDof9	AGTGGGTCTCCCTGGCGATA	AGGGTCATCAATCCTGGGTGTT
	MaDof10	GCAAAGATTCTGATTCTGGCTCG	CTGGCATGAAAGGGAAAGGGTG
	MaDof11	GACCTTTCCAGTTGCGTTCTACC	CTCGATCATGCTTGCATCCCTC
	MaDof12	CTCCAGCCATTGCCGTCCTT	GTAGTCTTCGGTTCGCTTGGG
	MaDof13	TCTCATCCCTCCATACATCTCGC	GGGTACGCTGACATCGGCAT
	MaDof14	ATTCGTGCCGATGACGCTGC	GCCCATCATCCCGTTCCAGA
	MaDof15	GGCTTCAGCAGATACAGCAGTTTC	TGTTGGCTGTCCTCCATCTTCG
	MaDof16	CTCCACAAGGAACCACCCAC	TTAGACCACTTGGGTTTAGGGC
	MaDof17	AGCGACCCAATCCCTCCAGA	TCCAGGATGGTCTCCAAGTGC
	MaDof18	AGAACAAGCGGTCCTCCCACT	GCAATCCGAATCCAGTAGGGTA
	MaDof19	CTGGCAACTTAGGCAAGCACTC	GAGAAGCCCTCCATTGCTAACC
	MaDof20	GCAAATCACCATCACCCTCTACG	CTTCCCATTTCAGGCTTGATACCA
	MaDof21	ACCTGTCATACCCTTCCCATTC	TCTCCACGGACTCAGAAATAGC
	MaDof22	GCCAACAACATCAGCAGCAGTA	CCTCCTCCGATGACACCGTT
	MaDof23	AGACCTCAACCTGGCTTTCCC	GACCACTGGCTTCACATCTTCAA
	MaDof24	ATGGCTGTCCCCTGTAGAGTC	ACCACTTCTTCTTTCTCCTACTGC
	MaDof25	CCGATCTACAATCCTCCTTCAACG	AGACCTATGTCACCCTCGCTCA
Y2H	*MaDof10-BD*	CATGGAGGCCGAATTCATGTCCGAGGTCAAGGACCC	GCCGCTGCAGGTCGACGTCATGTGCTTTCCTGAAAGGAATG
	MaDof23-BD	CATGGAGGCCGAATTCATGTGTTATCCTTGGATAGCTGCG	GCCGCTGCAGGTCGACGTCAGTAGAGACCACTTCTTCTTTC
	MaDof24-BD	CATGGAGGCCGAATTCATGTCCGAGCTCGGGGAC	TCAGTAGAGACCACTTCTTCTTTC
	MaDof25-BD	CATGGAGGCCGAATTCATGCAGATCTTCATGGAATCATCTG	GCCGCTGCAGGTCGACGTCACAGACTCAAAGGAAACCCC
Subcellular localization	*MaDof10-GFP*	CAAATTCGCGACCGGT ATGTCCGAGGTCAAGGACCC	TGCTAGTCATACCGGTTGTGCTTTCCTGAAAGGAATGGG
	MaDof23-GFP	CAAATTCGCGACCGGTATGTGTTATCCTTGGATAGCTGCG	TGCTAGTCATACCGGTCCATGAGCCGCCTCCACC
	MaDof24-GFP	CAAATTCGCGACCGGT ATGTCCGAGCTCGGGGAC	TGCTAGTCATACCGGTGTAGAGACCACTTCTTCTTTCTCC
	MaDof25-GFP	CAAATTCGCGACCGGTATGCAGATCTTCATGGAATCATCTG	TGCTAGTCATACCGGTCAGACTCAAAGGAAACCCCTG
BiFC	*MaDof23-PEAQ-YCE*	CAAATTCGCGACCGGTATGTGTTATCCTTGGATAGCTGCG	GCACGCTGCCACCGGT CCATGAGCCGCCTCCACC
	MaDof23-PEAQ-YNE	CAAATTCGCGACCGGTATGTGTTATCCTTGGATAGCTGCG	TGCTCACCATACCGGT CCATGAGCCGCCTCCACC
	MaERF9-PEAQ-YCE	CAAATTCGCGACCGGT ATGGTGAAGAGCAAGATCAGAGG	GCACGCTGCCACCGGTGCAGAAGCTCCACAGATGGAT
	MaERF9-PEAQ-YNC	CAAATTCGCGACCGGT ATGGTGAAGAGCAAGATCAGAGG	TGCTCACCATACCGGTGCAGAAGCTCCACAGATGGAT
Dual LUC assay	*MaDof23-pBD*	TCGCCGACCGGTAGGCCT ATGTGTTATCCTTGGATAGCTGCG	AACCAGAGTTAAAGGCCT CTACCATGAGCCGCCTCCA
	MaDof23-PEAQ	CAAATTCGCGACCGGT ATGTGTTATCCTTGGATAGCTGCG	AGTTAAAGGCCTCGAG CTACCATGAGCCGCCTCCA
	MaERF9-PBD	TCGCCGACCGGTAGGCCT ATGGTGAAGAGCAAGATCAGAGG	AACCAGAGTTAAAGGCCT TCAGCAGAAGCTCCACAGATG
	MaERF9-PEAQ	CAAATTCGCGACCGGT ATGGTGAAGAGCAAGATCAGAGG	AGTTAAAGGCCTCGAG GCAGAAGCTCCACAGATGGAT

Table S2. Related information of each *MaDof* gene.

Gene	Genome serial number and name	Chromosome number	Location	Size （bp）	Size （AA）	MW （kDa）	*p*I
MaDof1	GSMUA_AchrUn_randomT13230_001 Putative Dof zinc finger protein 4	Un_random	63755836-63756675	840	280	29.92	8.75
MaDof2	GSMUA_Achr6T10720_001 Putative Dof zinc finger protein DOF5.5	6	7136799-7138350	822	274	30.11	9.69
MaDof3	GSMUA_Achr6T32240_001 Putative Dof zinc finger protein DOF5.2	6	31725587-31726849	1170	390	41.97	5.29
MaDof4	GSMUA_Achr10T03660_001 Putative Dof zinc finger protein DOF3.6	10	11901841-11903032	873	291	30.76	9.3
MaDof5	GSMUA_Achr10T02500_001 Putative Dof zinc finger protein DOF3.6	10	7268980-7270119	1014	338	35.82	9.86
MaDof6	GSMUA_Achr3T10670_001 Putative Dof zinc finger protein 4	3	7878831-7879607	714	238	25.68	9.53
MaDof7	GSMUA_Achr7T15150_001 Putative Dof zinc finger protein DOF2.4	7	12143943-12144613	546	182	19.43	9.93
MaDof8	GSMUA_Achr5T07660_001 Putative Dof zinc finger protein DOF5.5	5	5496748-5497918	909	303	32.93	6.94
MaDof9	GSMUA_Achr10T18770_001 Putative Dof zinc finger protein DOF3.3	10	25356377-25358204	1074	358	39.41	7.96
MaDof10	GSMUA_Achr9T05020_001 Putative Dof zinc finger protein DOF5.5	9	3265215-3269729	1533	511	54.68	5.55
MaDof11	GSMUA_Achr3T01850_001 Putative Dof zinc finger protein DOF5.5	3	1251559-1252883	1041	347	37.84	7.12
MaDof12	GSMUA_Achr10T09190_001 Putative Dof zinc finger protein DOF1.4	10	19128892-19129938	1047	349	37.53	8.41
MaDof13	GSMUA_Achr4T27160_001 Putative Dof zinc finger protein DOF4.6	4	26111997-26112839	843	281	30.62	9
MaDof14	GSMUA_Achr8T11850_001 Putative Dof zinc finger protein DOF4.6	8	8590499-8591439	897	299	31.61	7.62
MaDof15	GSMUA_Achr8T02150_001 Putative Dof zinc finger protein DOF3.6	8	1595234-1596410	999	333	35.12	9.32
MaDof16	GSMUA_Achr11T10790_001 Putative Dof zinc finger protein DOF2.1	11	8546282-8547172	891	297	32.02	6.70
MaDof17	GSMUA_Achr2T15700_001 Putative Dof zinc finger protein DOF1.7	2	16888165-16888839	675	225	24.22	8.13
MaDof18	GSMUA_Achr2T09970_001 Putative Dof zinc finger protein DOF4.6	2	13296598-13298657	1299	433	47.44	9.23
MaDof19	GSMUA_Achr2T22000_001 Putative Dof zinc finger protein DOF5.2	2	21252239-21254499	1137	379	40.71	9.01
MaDof20	GSMUA_Achr11T25090_001 Putative Dof zinc finger protein DOF3.3	11	24332306-24335038	1266	422	46.08	7.14
MaDof21	GSMUA_Achr11T15580_001 Putative Dof zinc finger protein DOF3.3	11	17278217-17281065	1401	467	51.20	7.84
MaDof22	GSMUA_Achr3T18260_001 Putative Dof zinc finger protein DOF1.8	3	19744997-19745898	675	225	24.77	6.89
MaDof23	GSMUA_Achr11T03360_001 Putative Dof zinc finger protein DOF4.6	11	2438953-2439972	1020	340	35.99	8.87
MaDof24	GSMUA_Achr11T09150_001 Putative Dof zinc finger protein DOF5.5	11	7071572-7072496	837	279	30.89	8.43
MaDof25	GSMUA_Achr1T20550_001 TARGET OF MONOPTEROS 6	1	15472269-15473459	843	281	31.37	8.99

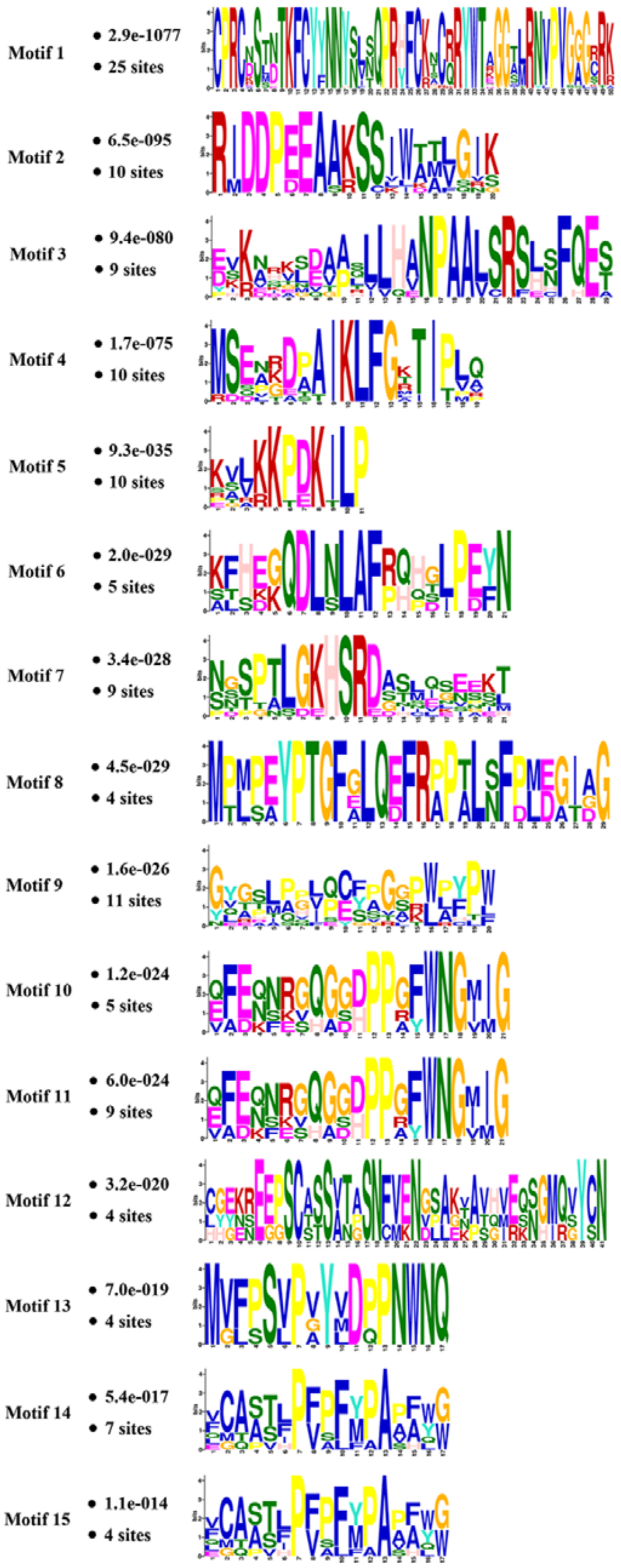

Fig. S1. Multilevel consensus sequence identified by MEME among banana fruit MaDof proteins. Motif 1 represents the conserved Dof domain.

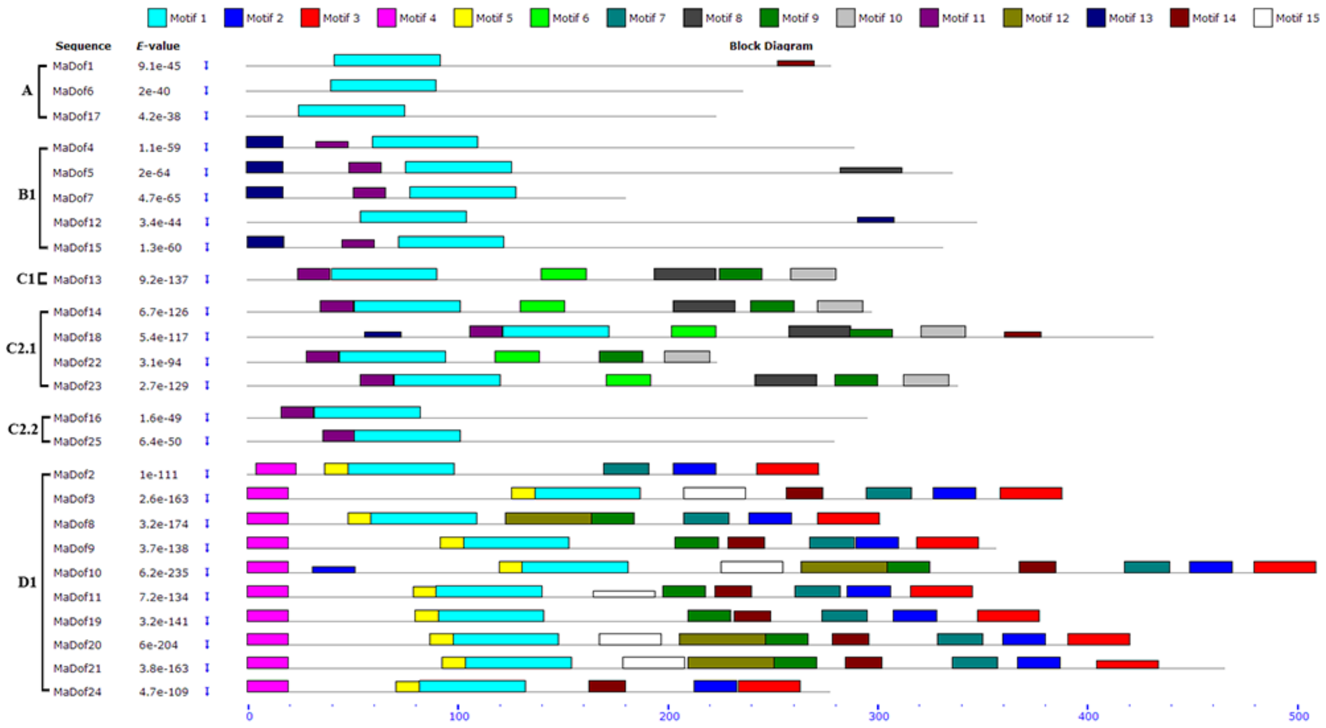

Fig. S2. Schematic distribution of conserved motifs in banana fruit MaDofs identified by MEME. Fifteen conserved motifs are shaded in different colors. Motif 1 represents the conserved Dof domain. Several subgroups were distinguished by the motif distribution, which is consistent with the phylogenetic analysis in Fig. 1A.

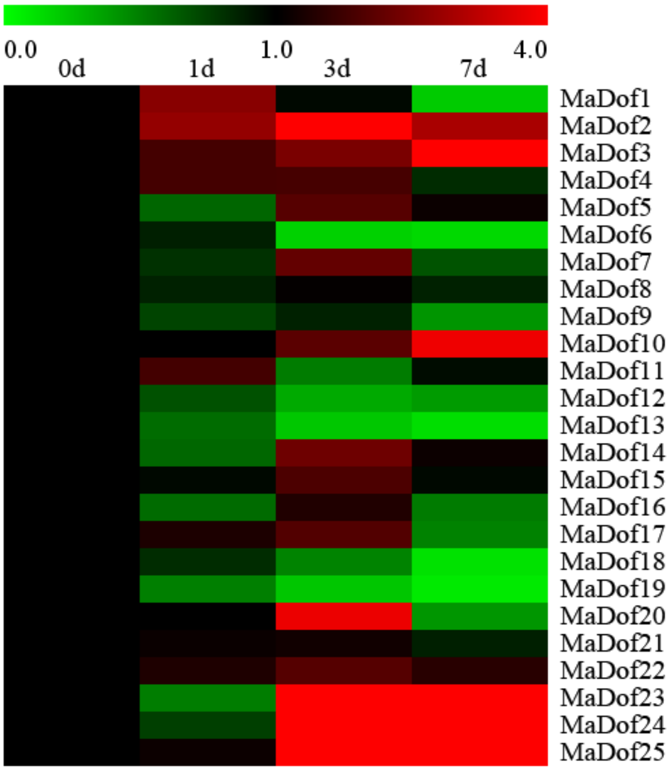

Fig. S3. Hierarchical clustering analysis of 25 *MaDofs* expression profiles in ethylene-treated banana fruit pulp. The cluster was generated using MeV 4.9 clustering algorithm according to gene expression profile analysis by qRT-PCR. Data are log2-transformed (or−ΔΔCt) value of gene expression of each time point compared to day 0. The scale indicates log2 variations: red, increase; green, decrease. The physiology data related with fruit ripening and softening, including changes in fruit firmness and ethylene production in banana fruit with ethylene-induced ripening has been described in Shan et al. (2012).

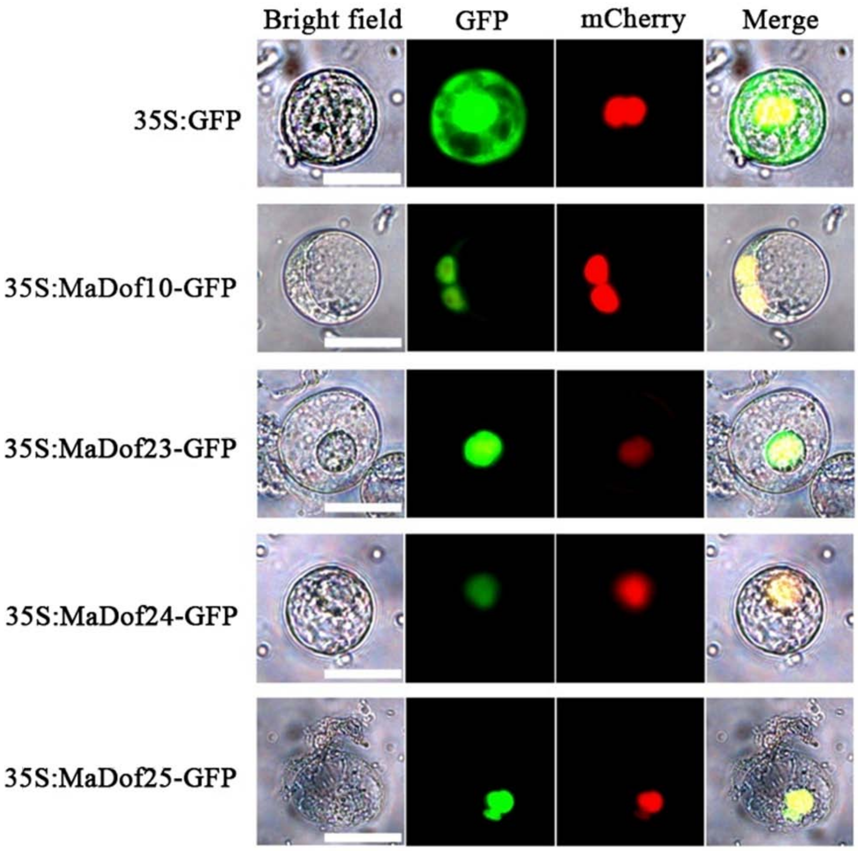

Fig. S4. Subcellular localization of MaDof10, 23, 24 and 25 in tobacco BY-2 protoplasts. Protoplasts were transiently transformed with 35S:MaDofs-GFP constructs or 35S:GFP vector using a modified PEG method. GFP fluorescence was observed with a fluorescence microscope. VirD2NLSmCherry was included in each transfection to serve as a control for successful transfection, as well as for nuclear localization. Images were taken in a dark field for green fluorescence, while the outline of the cell and the merged were photographed in a bright field. Bars, 25 μm.

>*MaEXP1* (GSMUA_Achr5G07470_001) promoter

CGCACAGCGTCCTCAAGCGGCCGCGTTTCTGCCTGGAGTTGGTCGACGACGACTAAATCTCTCACG
GTGGCGACCACTCGTGGGGACATGCAGAGGAAAAGGTTCGCGAAGGCTATCGTGGACAAAGCACT
TCTTCCATGTTTTTCTAGCTCACAAGTTTGTTGTACTCTGCTCGATCCGTGTTTGCACTTCGCACCGT
CTTCATGTGCTTGAACTCAGCTACATGAACGAGCAAATATTTCAAAGTGCCGGTGAATGATTGGCGT
TCTTAATCTGCCGCAAAAGAGTCAAAGACGGTGGGTAAGTTTGACCCATCATTTCCGACACTGATA
CTGCCGAGTACACGTACACTATTGCTTGCGGGATTTGCGGTGTGGCCATTTTCTAGTCGACTCGATG
CGTTCGAAGAGCGTCTCTGTCTCTGTCTTCTTCCTCGCCACGTGAAGACGTCGGCAGCCCGGAACA
GCATGTGCTCCTCCTCACATGGTGGGACAGTCCGTATTCAAAAAGACATTTTTTTCTGACTGTTTTC
TTTCACTCGTAGTGATTCATAAACTATAGCCATCTTCCCAGCATACACATTAGTTAAGGACCATAAGG
TCTTGCAACGCACACTAATGGTTTAATCTAATCGAACAGAGGAGGTGGATAGCAGCAACTCATTGT
CTAATGATTGCTCATCTTCCATCGTGTAGTGTTGTTTATTAGCGTACTGACATGATTGCGGCAATTAGT
TTGAGCGAGTCATAGTAGCTGGAGCGTAAAGAATGGAGTAATCTCGGCGTGATTGATTGAAGCACG
CATATTTGTGCCGCAGTATGGCACCGTCGGCCATACAAGTCCGATTGAGGCCCTTTATTTCTTGAACT
GCTTCGGCGCGCATCTTTGGATTTCCAGCTCCGATACCTATGAACACAGCCTTCAAAGAGTGAGTC
AACAATGTGAACTGCGCAACTTCTTCCTCTGCACGCCATTCACATCATCTTCCTCTGCTTCTGTTCCA
TCAGCGAGACTTATTTGCGCTTCCAACTAATCTTGTCTTGTCCTGCCCTCTCCCCATCTCCTTACCTA
TTGTCGAATGTAAAGAGAACCCCAAGAGAACACAACAGGATAAACGTGGCACCATGAAGTTTCTT
CCATGAAAGCAGATGCATTTGGGTGTGTAGTTGGTTTTGTCACCGGGACGATTGCATGTGCTCCTGA
TTTGGATGATCGCTGCGGTCGTCTTCAAGCCTATGGCTGGCCCATGGCCGCCACCAACTTGTCTCTT
TCCAGAGATTGAAGATGAAGAAACCAACTCCAACCCTCACTCTCAACTCGTATAAAGACACAGCCT
TTCCCTCCGCTCCTCTTCACTACTCCTCCTGTTTGCTCACTCACGACTCCAATCTTTCTTTAGTGGGA
ATAGTTCCAATG

>*MaEXP2* (GSMUA_Achr11G22960_001) promoter
CACCCCCTCCATAGAAGGTTGCCGAGGCAGGAACCACATCAAGCTTACCGCAGCCGTCTGAACTAT
GTCAGACGCGTTCTAAGATCGTTTTTCAAGGAGCCTCTTGCAAGCGACACGTGTCGTGTCGATGCA
CGAATCTAGAAGCATTTACCCTCCTAAATGTGAATGAATGTAAACAGAGTAAGAACTCGACGGACT
CGACCACATCGAGAATGATACTGTCCGGATTTATCTTGCGTCTTATACTCCACAAAGACTCGTTTGG
CTCATCGTTACAATAAATAGCAATACCTTTCTTTTCCTTTTGATTAATATAACAATCATTGGACCGATG
AGTATGAATGGCAGCATACTATGCAGGCCTCAGGGAAAAGTAACCCATGTAAGTGTGAATAGGATG
CATGAGTTGGGTTGACTTCCACTCCGCCTCGCACATGCACCTACGCACTACCTGAAACCTAACTTG
GTTGTGCCATTACGTGACGAAATAGTCAACTCCTCTCTCTCTCTCCTCCCCCACATGGGTTGGTTCA
TGAGGTGCTCCGGCATGTACTGCATACACGAAGTCTCACCTCTCACGCCACGCACGAGTCACAAGT
CATCTCCCTTTCTGTATCCAACCACCACCGTCATACTATTCTCGCATATGGACATGTGTATACGTAGA
ACATGTGGTTGCGATCTCGTCATCTACCATCCGTCTGCGTCCATGATGTACGGGAGCCATGTGCTTG
CAAACCCTCAGAAACTAACGACGAAAAGAATACTAGACAGAGCACAACACTTGGTTGACTTGATT
AAGTTACTTATGGTTCCGTTTCTCTATGCTTCATAAGGTAGAATGTACGGCTACGTACACCGTTTCCG
TCTTCGGGTGGAGGCGGCGCAAAAGGTGGCCAAGTGTAGCCGGCACCAGGCTGTACATTTTTGTAC
GAAGCTTGTTTGTATATAGCTTTGATTAAGCTTCACCAACTGGTAATAATTTGTTCCAGCGCGCAGGT
CTTATAGTTGCGTGAAGTCAACGTAGACTAAACGCCCAACACGCCGTCAGGGTCGAAGAAGAAGA
GCTCAGATTCGGCACTATATATAGGAATGAGGGAGGTGGGATGGTATGCATCAATCTTCACAGCACC
GAGGAGTTGTCTTCTTCGAGCAAGTAGAGATG

>EXP3(GSMUA_Achr10G26620_001) promoter

CGCACAGCGTCCTCAAGCGGCCGCATTTGTGCCCCCTCGACTTGCGTCTCTCCCATCTACCCTCTCT
CACGACTCCCCCTCCTTCCCTACCAGTACTGTTATTGGCCCTTGGCTTAACCCCACCCACGTCCGTA
AAATTAATCCAATCACAAGACAGATAGGCCGTGGGCTGACAGTCTCTGGCGTAACGCGACTTATTT
CAGTCTCCTTTTCTTATGCGGTAATAATAATAATAAGTAAAGTTGGATCTTAACGCCGACGGACACCG
TCGTCCACGTGGAAAGATGGGACGATGTCGGTCCCGCTCGAGGTTGACTGGCGCGCAAAATCTGG
ACCGTCCAATACGCGATGGGCCCACCGCAAAATCACGAGTTCGGTGGGTGAGATGTGAACCAAAG
CGAAAGACTAGCCCAGACGGGTGCTAATTTCCGGGTAATCGAACGTCGGGTGACTGCTTCTCTCCG
CCAAATCCCCGATTTTTGATTCGAAGGAAAATTATTCCCCCCTATTTTATTTATTTTCTTTTACGAAAA
TAAAAAGAGATCAAAAAGCGTGGATAAAAGGTCGTCTAATCAGAACCGTCGGTGGCGGCACAGCT
TGCGAGGGAAGCGATCCGAGTACGATTACAGTGCCCGTCCGATTTCCCACTTCACCGCGACGCAAC
GTTAGTTACCCGTGGCTCGCCCCGATAAAATCTCAGCCGTTGGTATCTCATCGTACCGACCACGTTG
CAGACACAGCTATGATGGGAAAGTGGTGCGGTGGAGCCCCCAATCCTTTAATCTTAGCAGAAAAGA
CTCTGAGTGCCATCGTTTGATCTCACACTCACACCTCCTCTGCTCGAGCCTACAAATACCCCACGCT
TGCCTCCCTCCACATCCCCATCCTCCAAACCCTTGATCGCGAGTGAGAGAGGAGCAAATG

>*MaEXP5* (GSMUA_Achr7G10570_001) promoter
CGCACAGCGTCCTCAAGCGGCCGCCTCGTTGCCTTTTTCTCCGATGCCCCCACGTGTCCCTTGTCGC
CCACCCGCCCAGTTACTGGAGATGCCTCGGGACCCACTCCTTTTGCTTCAATGCCAAGCCGCCTGG
GTGTGACCCACGACGTGTCGGTAAACCTGTTGATGGGTCCCTTTTCTTTCTTCGGCCCGTCCACCTT
TACAACGAACCGAGTTGATCGAGTTGGTTCAACAAAATAATACAAGATGGCTCGATGTGGGTCCGA
GTACACGGTCGGCCCGCCGAATCTGGGCCGATAACCACAGAACTGAGCTCGAACGGAGCCCATCG
CATATATATATATATATATATAGGTTTGAGACAGTGTTGTGTGGGAACGGTGTGCCGTAGGAAAGCGGAG
GCGAATCAGGGCCGTCGGTGGCGGCACAACTTTCAACGGATGCAAAGAGGAGGACTGCGGTCTTT
TGGGTCCCAAAGCAAGACCACCTTTCGCTCCCGTTTCCCGCTTCGTTTGCTTCGTTTGGGCGCGAC
TTCAGTTACGTTGCTCTCTTAGTAGACCAATCACGGCCGTTGGCTAATCATCTGACGTCTTACGTCG
CTGACGGTGCTATGATGTATGGTGGCTCCCCACCGATTGACTCGTATCCAGTGTTCCCACGACCCTT
CAATCAGAACGGAAGCGCCTCGCTGCCTCCTCCATTTCCGCTATTTACTTCCGCGAAGCGGCATTAA
TTACTCACACTGCCATTCTTCTCCTCGAGTCCATAAATAGCCCTGCTTCCCGTCTCTCTTCGGCCCCA
TCCCCCACACTCGAGAGCGCTAGCGAGCGAGATAATG

>MaXET7(GSMUA_Achr7T16430_001) promoter

GGTTTGGACCATAACTAAATTGACGTGTGACCTGATATGCATTAATCTTGATAAGATTGACCCAAAC
TTATAATTATTCGATTTCTGACAATCTATTTCAAGAAACAAACCTTAAAATACAAATTAATGTCACTT
CAACGACCAACAATAGAGAAAAAACATAGATAAAAGTATGAAAAATCTTTATATATTTAAAAATCAT
TGGTCACTTTATCAAATTCTCTTGATAAAAAAGATATAGAAACCAAACTTTATATTACTCGGCTTATTT
CTTTTTTATGATTAAAGTTATCTGTTATGCCTCCAAAATGGTCTGTGGACTAATGATTCAGGTTTTAA
AGGATAAAAAGTTTTGACGTATTAATGATTAAAACACGTTACATTGCATTTTATTTGATGATTATGCTA
TTCAAAACCTGAAAGAGAACTCATTAAGTTTTCTTCAAGTAAGAAGGTAATATGATCAAAATGTGA
CTCGAGATGAAAATCTGCATGTTCTAATCCGCACTACCAAAAGGAATTAGAGCCTGCCAAATTACA
AGGGAGAGCGGGCCGAAGGATCTTTTCCGCATTGATTAGGAAGGCAAGCAAAATATAACTCACCCA
TGACAGCCTAAACGCACTCGGCGTCATAGCTTAATACATACCATCCGGCCATGCCTAGATGCGGCTT
GAGATCGCAGAACGAAGCTCATGAATGCACGGCAACGTTCTAAAGTTTCCTCTCCATGGAGGCAAC
TCCTTTAGTTGGAAACCCCCTCGTCACTTCCTTATCGCCCGACAACGGCATGATCACAACGCGTCAC
GGTCAACGTCGGTCTCTAAAAACGACACTCTAACAACAGCTGGAAAATCCAGCCACCCCGATGTCT
CTCCTCTCACCCAACCCTTCTACTCTGCACCCGCCGACGCTACTGGCCTTGTGATTGAACGGACGG

CCGGGACCATGGGTCCCAACTGCTCTAAGTGCAAGGGCAACATATAAACCGGTGGTGAAACGTAA
CTTCACATTGTTGTAGTGCTTGCCATGCACGCTATAAAAGAAGAAAAATGAGCTTTGTGGGGATTGC
ATCGTACCTGATGACGCCACCTGGCCAGTCGCATTCAAATATCGGAGGAGCCGCCCGCTTTAGTGG
ACCATGATCACGAGGCTTTCGTCCGAGGCCGCTCCACCACTTGAAACAGCAGCACCATGGCCGCGT
CCGCCGACCCCGCGTCTCCTGCACATTGCCAACCCATAGATTATTATTTAGCATACAGCAACGCCAC
CATCTCATCTGCTATATATAACGCCCCAAACCCCATTGCTTACATCGTCATCTGCTTCCCCCTCCTACG
AAGCTTGTTAACGTTTCTGCTATTGCATACCATGGCAATGAGGTTTTTTTTGCTGGCTTGCTCCTTGG
TCGCCGTTGCCTCCGCTGGTAACTTCTACCAGGACTTCGACATCACCTGGGGCGACGGCCGTGCCA
AGATCCTCAACAATGGTCACCTCCTTACCCTCTCCCTCGACAAGGCCTCCGGCTCCGGCTTCCAGTC
CAAGAACGAGTATCTCTTCGGCAAGATCGACATG

>MaPG1(GSMUA_Achr3G13670_001) promoter

GGAGATAAAAATTTGTTAATTTGCATGATGTAAAACTTGCTAAAAGTGTTATCTTGCAAATCTGAAG
AGAAGTAATGTTTGCTAGCTTACACATTTCAAGAAGATACAAAACATGATATCTTGCACTTCTCAAA
AGAGAAGAAAGAATTCATTAGTTTGCATGTTCTAAAGTGATGAAAAATTTGCTAAATAGTTAGGCTT
TCATATCTCTAAAACTTGATAGTTGTACTTCTTCTTTTTGTTGATGACAAAGGGGGAGAAGATATGAT
GATATGAGAATCATACATGGTAGAATGTAATATATTTTGATATTTTAATATCATGATGATTTGAAATATT
TATGATGATGACAGCCTCAGAGATTGAGTTTTTCGAGTTTTCCAACTCACAAGCGGTTCCACCGCTT
GTTCTGGCGGTGCCACCACTTGACCCAACTTTTGAATCACTGAATGAGCCTCCCAATGAGCCCAAA
TCAGTCTCAATTAAGACCCAATTGGCCCCTAATTGAGTTAGCATGATTACACCAAAAGCTAACTCAA
TTAGCCCCCTAAACTACTTCGATCTTAGGCAAATGATTACAAAGCATGAATCTTTTGTCCAGCATGTC
ATTGGTTCATCCGGCGCTCGTCCAATCTTTCGACGCATCATCCTCTCCTTAGGCATATTACCCAATCG
GCATGTTGACTCCCGTAACTTTCGATCTCCTTAGTGCAATGTCCGATACTTCGGCCCAATGCCCGAAT
TCACGGCATAAAGCCTTCTGCGACACGTCGATCGATCCTTCGGCTCGACGTCCAATCTTCTAATATG
TTCACTCCGGCCCAATGTCCGATTCCTCCTGCTTTAATCAATTTGTCTCTACTTGATCGAAGCTAGTT
CTGCGTCACTCAAAACACAGATTAGATCACAAACTTATTAACTAATTTCATCATCAAAATTCGAGAT
TCAACAGTAATAACATTCCTTATCCTTGTGACACCATCTATATGAATCTCAAAATATTTATAAATCTTT
CTTGTGGACTTATTCTCATGGCAATATTTCTTATCCCGGTGACAATAGTTTTTGAAATTTTAAAATGCT
CACTAGAAATGAGACAACGCTTGTGCAAGTGTTTGATTTTGCTCTTTCTTATTAAGACCTTTTTTTTT
ACTTAAAATAAAAATAAAGAGATTTACTCGACGTAGAAGATGAGAACAATAGTTTCAAATCGAGTG
CTGCCACGTGTAATAAAAGAGCTATCCCGCAACAACGTTGCAGATGCACCGACCTCATTCAATTCGT
GGGGAAGAAGAGGACACGAGAGGAGGAGCAGAGACCGGTCATCCTCAACTTAATCGAGCGTCAT
CGTGATGGGCCGAATCAAGCGGCGAATCAATCGTTTTCCACGCGGCGGTGCACTTGCGTCCGATGC
TCCTCATCTATCATCCGTCCGTCGATCCAAGCACCGAGTACGGATCCCATCCCCTCCCCATCTGCGC
AACTTCGTCGACGGAACTGCCCATATTGATGGGTCTCTTCCGCCCGATGGAGTACCTGCCTGAGCTA
ATTTACAGACAACGGATGTTCTTTCCTCCCTTGGCTCGCTACCCCTCACAACCGCAGAAATCCCATC
CCGACCCGAAGTTGTGGTGGTGGTGGCCATG

>MaPME3(GSMUA_Achr11G20560_001) promoter

TAGCTCTGTCCGATCCGCTTCGGACGGTGAGCTGACAAGTCATCACCAGCTACACACCTTTCCTTCT
TCTTCTTCTTCTTCTGCCGACCTCGTCCCACCTCCGAACTGCACATCTTGACTCCATTGTGGAAGTG
TCCCAACTCTGCCTGTCTTCATGCAGAAAATAGCAGCTTATGTATTCCCTTCGTTGGTCTCTTCGTCT

CTTGCTTATTAGTCTTGGAATACATGTGTGTGACAGCCTGATGCATTATGATTGCAAAACCGTGTTGC
GATGCATCTTTTCCAGCTTCCAGTAGATAACATCAAAATGTACCTTTGTATTTTTCTTCATGTTCTCAA
TATAATATTTATTATTTTATTGAACATCTGACATAGGTTTAATTAGAATCATAATTGACTTCGATCGTAA
TCAGTTTGACCGATTCAATCTCAGTTCGGCAATTAGCCGAACCGGTACGACGGATTGGGTTCGAAA
AAGTTGGTCCCAAACCACGAAGACTACGAGGATACAGCTGCGGTGGTCAAAAGATGTATGTTAATT
GTCTCCTCTCCTCGATTCTCGCTATGACTTGTGTTCTTACGTTTCTTCAATGTCAAAACTACAGAGAA
ACCACATCTAAGATTGAGCTTTTTGTCATCGGTCCAGATTTGTGTACTTTACTCGGCAAAAGCTGAT
TCACCCTTCTTAATCGTTAGTCATTCTATTTCCTTCTCTATAAACATAAACATGTGTACTCTCTCTCTC
TCTCTCACTTCCTACTCGACGCGTTTCATTGGTCTCGAAAGTCAACAATGTTGACCTAATTAGTCTG
ATATATCTCATATGTTATGCTGATCGGTGTAGTAGTTTGCAGCGTGGTTTGACTTCCTCAAGAATGCG
AAGAACCATTTCCCTTGGAAGGAGTGCGAGAAATGACCACGAAGACCCTCGAACCCTCCTATATAA
CTCCCCTGTGACCCCTCGCCACCTTCGTCGTCCCGAGGAAGAGCTCTTTACGACATG

>MaPL2(GSMUA_AchrUn_randomG04250_001) promoter

GCAATAACTCTTCCATGACCCGAACGCCGAAGATAACACCTGGAATTCCACGAGTTCTCGGACACG
ATAATGCATGGCAGTGAGGAAGGAGAGTTTTCTTACGACACAGCAGTCTTGCATGGATTCTATTGGA
ATTCGGAAGTATCCAGTAAAAAGCTGTAGTCGATTCGACCCTAAATAATAATAATAATAATAATAATA
ATAATAATAATAATAATAATAATAATAATAATAATAATAATAATAATCAGAATAGTAACAACAATAA
TTGTAGCACTCAGAAGGCAATTGAACTTTCGTATCACCATTCGCACCTGCCAAAAATGGAGGTCGC
ATATCTGTGTGCTCTTGCGAGGCGGCTGGACTTTCCTGCACGCAATCATGACTCCATCTCCTCCCTTT
ACGTGAGATGGATGGCAAGATCATCAAGTTCCAGTTCTCCACCTTTCAATGTGGCACGATCAATTCG
ACTCGGAATAAATCTGATGTGCCCCATGCGATATTTCTTGCCTTGTTTCGGCGAGAAGACAACCCAT
ATGCTCCTTCTATCAGAAGATCGATGCACATAGATACTCATCACAGCCGGGATATCAAATCTGATATA
ACAAGAAATCGAGGCTGAGAAGCGTCAGATATAGAAAATTATGAGGCGACACTCACCCACTCTCGG
TGTTCAACGATCTTGACCTTCTTCAAGAATCAACCACTTTGATTTGAGAGCATCCCTCAATCAATCG
GGCGGTCAAAGGTATTCAATTTTGCCGACTGGTGTACGATGGACATGTTCAGATACAGCTCAGGCT
GAAATGGAAACGGATGACGAAGATGCTATGGTCGTCAAGTGCGTTTGCTCCGAGTCCGACTCGTGT
ACGAGAGAGAGAGAGAGACAGGGCACATGGGATGAGTGTTGACCGGTCCATCGGATTGATCTCAT
CGGATCATATGTGCCGACGGGTGAACGGAAGGAACAAGGACGAACATGAACCTTCCTTTGTCTGA
GGTACTCCGGCGTACGTCACGCTTCCGAAGATGCTTGGACGCAGTGGTAACAGTTAAAGCAGCAGA
TGATGGATGACAAACATGGTGCAGTGCTTGTTCCGAGCGAGATAACCATTTGCACAGAGACCGGGG
GTTGAAGTTGCTGCCTGGAAAGTTGGGTTCCATGGCATTTGGATGGAAGCTTTGACCGTCACTGGT
TGACATTAACAAAGGCCATCGAGCATGCCCACTTCTTTCACTTTATCACTTTTACTCGAAGCCTCTC
GTTTAGCGGAAGACTTCAAGATGTCAAATCTGATGTAAAGTTGGATTTCCATCATCTGGATTAAGAA
CAACCTCAAGTGTGTTTGAATTCTCTAAGCACCGAGCGCACGGCATTTTATCAAACGAATGGAAGG
CACACTCCATCGTATACTCCCTTGCGTGTCAGCCATGTCTATTTCCAAAAACTGATTGTTTATCAGCC
GTCTAAAACGCATCCGATGGTCGCCATCCAAATTGTCAAGTGCGGGGAGCGGCGTCGACGTCGACG
TCCGCCACGATAACGCTCGTGGGCGAAACCCGGTACGACGTCCGACGTGGACGGGCGGCCCCCGC
TACGGATGGAGGAGCGAAGCTTCTGTTAAAGCTGGTGTTGTTGTTAGTTGAGGAGGCGAGGAGTG
GTGTTCGTCGGGGCCCGATGCCGTCTCCGTTAACGGTGGGTTAACCATGGTTAGGCCGGGTTACGG
TGACAGCGAATCGGCTTCTATATAATGCCACTACCTTTGCGGTGTTTCCTTGCAACTGCATTCTCCAT
CAGCTTTTCTGTTCTCTCCTTCGATCACATCTTTTTGCTCTGGGAAACGTGAGAGGTGAGACGAGG
GCGGCAATG

>MaGAL(GSMUA_Achr7G08970_001) promoter

CAAAGCATTGGCAGATATTGGCCTGCCTATAAGGCATATGGTTCTTGTGATTGGTGCGATTACAGAG
GGACATACAACGAGAAGAAATGCCAGACTAATTGTGGGGAGCCTTCTCAAAAATGGTAATGAAAC
ACCTGCCTTTTTGTCCACTTCAGTCAAAGCATGCAAAAGAATCGAATAAATGACTGATTACTGACCA
CAAATCTCAGGTATCATGTTCCTCGCGCTTGGCTGAACCCAACTGGAAACTTGTTGGTTGTGTTTGA
AGAGTGGGGTGGTGATCCAACTGGGATTTCTTTGGTGAAAAGAGTAGCACTATGAAGAAGCTTGCT
GTCAAAACCATCGGCAGATGATAACCACAGATGGTAGACCAAGTAAAGAAAATGAACTAAAGTCA
ATTTATACAAACCAAAGTCGTGGAGTAGTGATTGGTGGAGATGCAGGAACTGCACACCCACTTACT
TTAAGGTATTGCCTGGTTACGTGAGTAAATGTGCATTGAAAATATTGGATGCACTCAACATATTACTT
ACTGCACGTGGGACTTTGCATATATCGATTCCATGTGTGGACATAATATTAGCAATTGGGATAGCATG
TTTACACAGAATGGTTGGTCTCGTAATTGCAAGAGCTAAGCTTTTGCTTTTGCACTGTTTAAGCTTAT
AAGCATTTTGATGGATCTTAAGTGACCACCCTTTGAGACTTTTCTTTTATACAGGATCAATTAATTTA
AGCCCAAAATTTCAATAATATAATATTATTAAGAATATAATTCCTATGTCTACGGGGAAAAAAATTTCC
AATGAAACTTTCCAAGATAAGAAAAGATTCGATAATACACTTATAATTCTCTCGTTTTAAATCTTTAT
TCAAACTAAAAATAAGATACTGAGACCGAGCTCGTAACCCATATAAAAAGATTATATCCAGAGTAAT
TCTTAATTTTATAATAAAATTATATTATAGATATATTAATAAACCCGCTTACTACCCTAATATCAACCTG
GAAAAGAAATTGATCCTGAGCTAAGTTAAGTCTGTATATTGATATACTAGACAATACAAAAAATATCA
TTACAATAAGCTTTCTTACAAAATTAGCATTGTTAGTTTCTAATAATGTTCAACATAACCTGCCTATCC
TAATTTTCTTTAAATAGTAATTTTTAACAATAGATTTTACTCTTCCAACCATTGCCAACCATCGAAGTT
TCTCTTAACTGCCCAAAGGAATTGGAATCATAATTGATCACAAAGGAATGATCAAACGAACAACGT
TTTCTTTTTCTATTTTTTGGCTGCTGCTGTTCACGGGGAACGGAAGAAAACGTTGACTGGCTGGCGC
TGTGGGACCCGCGTACTCCGGCGCCAATCCTGTGCTCGTCTTCGCCCCTGCGGTATTTAAACCGCAG
AGGCACCCACGGCTCCCGCGCCGCATCCGTCCTCTTCCATCCCGCCTCTTTGATCTCCCTCTCTCTT
CCTCGGTGTGTGCTTGTTACGGTAACGCTACTGCTCAATAGTTCCTTCAGAACTTCTCTAGCGGAGG
AAAAGACGGAAGAACAGCGCGCACGAATCTCCATCATTCACATATTATACAGCCAGAACACGAAAG
GAGACTCCTCTGAAGCGAAGACCGACCGGCCACCATG

>MaCAT(GSMUA_Achr8G05090_001) promoter

TCATGCATGGTCAAAGACCCTCTGTTTGATCTCACCTTTTGGCTTTCACCGTAGTTACTATCGACGTG
ACTCCATGGAGAGATGTACCCTAACCACGCGTTAATGGACCATGGCATCTCTCTAGAGACTCCCCCA
CTACAATCGTGAAACACGGAATTAGCAAAAGCAACAAGAACAAAATACAACAAAATCTCCTAATTA
ACATCTAATTCCGGGCTAACTTTTAAGTTAGTGAGTGGTCGAATATAATGAAGTCATTTAATTTACCC
ATAAATTAATATATGGAATCGATGGTGGTCTCCATATTTAAGAACCCATAGAGTCGAGAGATGCAAGT
GAAACACAAAGCTAGTGCAACTTTTTCTGAAGGAAGAAAGAGAAGAAGATGGTAACAATCATTCT
CGACATCTCATTATAGTTTCATTGTTTTAAGCTTTGGTTTATGTTCGAGTTTAAAGCATGTTATATTATC
ACTTTTATCGAAAGTGTTAGCTAACCTAACTCAGTCGAAAAAGACTTCGATATATCGCATAATCTTGT
AGATAGATTATGATTTTTGTTATTAGTTATGGGTTTGACTTGTGTAATATCATATTTTCTAATTATCGCT
AATTAGTCGTATGGTTTTTTATAGAAAAGGTAGTTGATTTCGATCTAATAAGGCAAAAATTAAACTAG
AAAAGTTATTTATTCATGAATGAGTAATTGAAATTACTCATTAAGTCTGTGCTCGATATGGATTGGATA
TTCACGTTGTGTAGTGGTTGTAGGGCAGCCCATTGACTCGGCCCAATTGAAACTTCAATGTCAATGG
GCTAATCGACCGGGGTTAGGATGTGATTTGATCAGCCCGTTTAGGCTCCCACAAAGATTGGATCGAG
CCGGTTTTAGATGCAAAGCTCGCTTTCACCGAATTGGTTGCACCAAACCGACCAAGAGAAGAACA
CCACTTTAAAATTCGTGCTGACATGTGGGTTGACTTGTCAGCTCTCGGCACGTTACCGGTCGCTAGA
GACTGCCTTCCTATTGGAGGAAGACATGGGTCCCACGTCCTTCAACAGACCTATGAAAGGACGCCA
CCTGTTTGCTCGCCCGTCCAAACCATGAGAAGTGACGCTGAGGAGGTGTGGGACCCACTATTTCTA
TTGACCGACGAGGAGGCGTCCGTCCCAGATGGAGCGCATGCGGTTCGAATTCGATGATAGCAGGTT

ACAAGCGCGGTGGGAGAGGATCCGCCACTAGGTTAGTAACGGCTCCCCGAAAGTTATCGATATCGC
AAAACCGCACGCCGCCACGGATCGCAGAGGAGGGTTTGTTTGAAGAACGACGTCCCTCTGCTTAC
TCGAAACCTTCGAGAGGAGAGCCGAACAAGCGAGTGTTTGTGTGTGTCGGGGAGAGGGGATCGG
AGATCGAATG

>MaPDC(GSMUA_AchrUn_randomG11220_001) promoter

CTTAAGAGTCGAATAACGTAAAAAGGTATGACCTTAAAAAAAAAATAAAAATTTGAAAGCACTAAA
AAAAAGTATTTACTCACATGATAAAATATTTTTTTGTATTTTCTTAAGAGTCAAATAACGTAAAAAGG
TATGATCTTAAAAAAAAAATAAAAAATTAAAAGTAGGAAATAAAAAGTCGCCGGTGCCCTTTGTAAAT
AAGTCACTTCCGTCTTTGACCCGGCTTCTCCAGCCGCGCACCCATAGGTTGCTTGGCCACCCCTATT
CCACCCTCCCTGTACAAACCTCGGGCGACCATCACAATCATCATAACCAAGAATCCAATCTGCAGTC
CAGAAAAAAAGAAAATTGCATGGGCAGGAATCCGAGATGTTGGCCAACAGGTCCTCCCATCCAAC
TGTTTTTAGGGATAATCAAAAGGAACCGCCAGTCGCCCAGCTATTGGTACACGCAGACCACAGACA
TCAGGACGGCGGCGATTCGATCGCGGACGAAGCGGCTGGCTGAGATGGCCCTGCAAGCTTTGCGT
TGGTGTGGCACATAGTACACAGCTCGCTCGCTCGCTCCCAAACAACACAAACGTCCACCTGCAAAT
GAATGAAACCGGAGAGCTGCATTCTGCTGATGTGTTCGCCGCCGGATGCCTGCGAAAGTCGTTGAA
AAGTATTAACACTTGGAAGTCAGAGGAGACGTCGTAACACTATTCCATCCGACGGCTAACAATTAC
CTGCTTCCGCGAGCAAAGACCGGTTTGCATTTCTCTTTCCTCAGGCTTGTTATTTTATTGCCTGCAGT
TGGCTTCGTCAGGTGCGCACCCACGTGCTCGTGGATAATCTAGTTTATAATCGCTTCTGTATAATTGA
AGTATATCTGTCACCTAACGCTGTGTAGTTTGTAAGCTTATTAGTTCGCTTCTAGACGACGCGTCGGA
TGGATATTCCCAAAGTCCCCAAATGCATACATAGTTTGCCCCTGGTTTGGGTTCTCCGGTATCACAGT
GCGTGTTCCTGCTTTCGGACCCCAGTTTTCCCGTACCCCATCCCGTGTTCCTGCTGTGAGACCACTG
ATTTCCCACCCCGTCCGCTCTTCTCCTCGCCCTCCTCTTCCTCTACTTGCTTGTAAATAAGAACCTCG
CTCGACTCCCCGCATCCCTCCCACATCACAACCGGCGAGCGACACCTCTAACAAACAAACAAGCA
AACACCTTCTCTGCTCCCACCGCAACTACCGGCAAATG

Fig. S5. Nucleotide sequences of the promoter of the 11 ripening-related genes.

GCC box is indicated in red box, AAAG is marked by red. Translation start site (ATG) is shown in yellow box.
